# Multivalent fusion protein targeting VEGFR2 and DR5 receptors: assessing the antiangiogenic and antitumor effects via multimodal microangiography

**DOI:** 10.1186/s12967-025-06859-8

**Published:** 2025-08-21

**Authors:** Irina N. Druzhkova, Anna G. Orlova, Anastasiia S. Fedulova, Arina V. Avakiants, Alina A. Isakova, Ekaterina V. Kukovyakina, Yuan Zijian, Ekaterina A. Plotnikova, Galina V. Trunova, Andrey A. Pankratov, Anton A. Plekhanov, Alexey A. Kurnikov, Pavel V. Subochev, Alexey K. Shaytan, Marine E. Gasparian, Mikhail P. Kirpichnikov, Dmitry A. Dolgikh, Daniel Razansky, Anne V. Yagolovich

**Affiliations:** 1 Privolzhsky Research Medical University, Nizhny Novgorod, 603081 Russia; 2 A.V. Gaponov-Grekhov Institute of Applied Physics of the Russian Academy of Sciences, Nizhny Novgorod, 603950 Russia; 3 Faculty of Biology, Lomonosov Moscow State University, Moscow, 119234 Russia; 4 Shemyakin-Ovchinnikov Institute of Bioorganic Chemistry of the Russian Academy of Sciences, Moscow, 117997 Russia; 5 P. A. Hertsen Moscow Oncology Research Institute - branch of the National Medical Research Radiological Centre of the Ministry of Health of the Russian Federation, Moscow, 125284 Russia; 6 Institute of Pharmacology and Toxicology, Institute for Biomedical Engineering, Faculty of Medicine, University of Zurich, Zurich, Switzerland; 7 Institute for Biomedical Engineering, Department of Information Technology and Electrical Engineering, ETH Zurich, Zurich, Switzerland

**Keywords:** VEGFR2, TRAIL, Anti-angiogenic, Molecular modeling, Optoacoustic imaging, Optical coherence tomography

## Abstract

**Background:**

Anti-angiogenic therapy is a clinically validated method for cancer treatment. It was previously revealed that concurrent targeting of angiogenic and death receptor signaling pathways by a multivalent DR5-specific cytokine TRAIL variant DR5-B genetically fused with the effector peptides, SRH-DR5-B-iRGD, enhances solid tumor suppression and prolongs survival. The SRH peptide is aimed at blocking the tumor neoangiogenesis by preventing activation of the VEGFR2 receptor, while the iRGD peptide interferes with the activation of integrin α_v_β_3_, and enhances the tumor penetration. Here, we investigated how the antiangiogenic activity of the SRH-DR5-B-iRGD fusion protein contributes to its antitumor effects.

**Methods:**

An integrated approach has been applied involving molecular modeling of SRH-DR5-B-iRGD binding to DR5 receptor, optoacoustic (OA) and optical coherence tomography-based microangiography (OCT-MA) imaging of the vessel networks in xenografts of human glioblastoma and pancreatic adenocarcinoma in nude mice, supported by immunohistochemical (IHC) staining for vascularization marker CD31, and in vitro and in vivo bioactivity studies.

**Results:**

Molecular modeling has demonstrated that genetic fusion of DR5-B with the SRH and iRGD peptides not only enables the engagement of additional tumor targets VEGFR2 and integrin α_v_β_3_/NRP-1, but also improves the interaction with DR5 receptor. OA imaging of the vessel network in xenograft tumor nodes of human glioblastoma and pancreatic adenocarcinoma displayed a decrease in the vessel fraction in DR5-B-treated xenograft tumors, with the effect being even more pronounced in SRH-DR5-B-iRGD-treated tumor nodes. This data was consistent with the reduction in the number of perfused vessels in DR5-B and SRH-DR5-B-iRGD-treated tumors as quantified by OCT-MA, and also correlate well with the data obtained by IHC staining and tumor growth inhibition.

**Conclusion:**

Ameliorated interaction with the DR5 receptor and imparting antiangiogenic properties to the multivalent fusion protein SRH-DR5-B-iRGD resulted in improved antitumor activity compared to DR5-B. Thereby, SRH-DR5-B-iRGD can be considered as a promising candidate for the treatment of vascularized solid tumors.

**Supplementary Information:**

The online version contains supplementary material available at 10.1186/s12967-025-06859-8.

## Introduction

It is well established that neo-angiogenesis promotes rapid tumor growth. Anti-angiogenic drugs have long been approved for the treatment of a wide range of solid cancers. The most common anti-VEGF/VEGFR drugs, such as Bevacizumab, Aflibercept, Ramucirumab, Sorafenib Sunitinib, etc. are included in many therapeutic regimens for treating advanced and metastatic cancers [1]. However, antiangiogenic therapy has limited effectiveness, since it mainly suppresses tumor blood supply without eliminating malignant cells. This also leads to hypoxia and inflammation, thus increasing tumor resistance to chemotherapy (reviewed in [2]). Combinatorial approaches are therefore needed that target both malignant tumor cells and the tumor vasculature.

The cytokine TRAIL (TNF-related apoptosis-inducing ligand) is well known for its ability to selectively eliminate tumor cells by apoptosis, which makes it a perspective candidate for drug development [3]. In addition to antitumor activity, it is also involved in modulating endothelial cell function and vascular remodeling [4]. For example, recombinant TRAIL induced apoptosis and inflammatory gene expression in endothelial cells [5]. The oligomeric form of TRAIL induced apoptosis in tumor endothelial cells in vitro and collapsed blood vessels, thus reducing tumor growth in vivo. Importantly, the mechanism behind vascular disruption and antitumor activity suggested that TRAIL death receptor DR5 was expressed on tumor endothelial cells, but not on malignant tumor cells [6]. This establishes a specific role of DR5 in exerting anti-angiogenic functions by TRAIL.

Previously, we have developed a receptor-selective TRAIL variant DR5-B, which specifically targets DR5 and selectively eliminates tumor cells without significant side toxicity [7, 8]. To further improve the DR5-B antitumor efficacy by engaging additional tumor targets, we have recently engineered a multitarget fusion protein SRH-DR5-B-iRGD, containing VEGFR2-specific peptide SRH at the *N*-end and integrin α_v_β_3_/NRP1-specific peptide iRGD at the *C*-end of the DR5-B protein. The designed SRH-DR5-B-iRGD fusion demonstrated enhanced tumor inhibition and prolonged survival [9].

Considering that both DR5-B and SRH-DR5-B-iRGD may exhibit dual antitumor and anti-angiogenic effects, in the current work, we have applied a range of state-of-the-art techniques to investigate the contribution of SRH and iRGD peptide moieties to the anti-angiogenic and overall antitumor activity of the SRH-DR5-B-iRGD protein. Molecular modeling revealed the specific features of interaction of SRH-DR5-B-iRGD with the DR5 receptor, as well as of SRH and iRGD peptides with their respective target receptors VEGFR2, integrin α_v_β_3_ and NRP-1. The antitumor activity was investigated on two xenograft mouse models of human glioblastoma U87MG and pancreatic cancer MIA PaCa-2. The anti-angiogenic activity was studied by a set of complementary techniques.

In order to evaluate prospects for the therapeutic application of DR5-B and SRH-DR5-B-iRGD proteins, we comprehensively compared their anti-angiogenic properties with in vivo optoacoustic (OA) [10] and optical coherence tomography angiography (OCTA) [11]. The in vivo findings correlated with immunohistochemical staining of tumor node sections for vascular marker CD31 [12, 13]. Consistent results were obtained in xenograft models of human glioblastoma and pancreatic cancer in nude mice.

## Materials and methods

### Molecular modeling

**Model preparation**. The initial structure of the monomer SRH-DR5-B-iRGD fusion protein was constructed with the AlphaFold2 program [14] using concatenated sequences (SRH, linker 1, TRAIL, linker 2 and iRGD) and the TRAIL X-ray structure as a template (PDB ID 1D0G [15]). DR5-B specific substitutions (Y189N, R191K, Q193R, H264R, I266L, D267Q, D269H) [7] were then incorporated into the structure using the FoldX program [16]. In the PyMOL program [17], the iRGD peptide was edited in a ring geometry to create a disulfide bond between Cys295 and Cys303 by manually changing the ɸ, ѱ dihedral angles. The trimeric structure was generated by aligning the generated SRH-DR5-B-iRGD monomer structure to three subunits of TRAIL in the 1D0G structure. DR5 segments from the 1D0G structure were merged into the SRH-DR5-B-iRGD trimer to obtain the (SRH-DR5-B-iRGD)-DR5 model. Alignment and merging were performed using MDAnalysis [18]. The numbering of the SRH-DR5-B-iRGD residues was done to match the DR5-B numbering with the TRAIL numbering in Uniprot. The three modelling systems made with and without DR5, with or without SRH and iRGD peptides, are elaborated in Table 1. All models were energy minimized using the FoldX program [16].

**Table 1:** Description of modelling systems

Modeling system	Description
SRH-DR5-B-iRGD/DR5	Trimer of bivalent fusion SRH-DR5-B-iRGD in complex with receptor DR5 (derived from PDB ID 1D0G)
SRH-DR5-B-iRGD	Trimer of bivalent fusion SRH-DR5-B-iRGD without receptor DR5 (derived from PDB ID 1D0G)
DR5-B/DR5	Trimer of DR5-B in complex with receptor DR5 (derived from PDB ID 1D0G)

**MD simulations and analysis.** All-atom MD simulations were performed using the Gromacs 2021.4 software package [19]. Simulations were performed in the AMBER14SB [20] force field with parmbsc1 [21] and CUFIX corrections [22]. The models were placed in a dodecahedron simulation box so that the minimum distance between the box and the protein atoms was 2 nm. Na + and Cl- ions (to neutralize the system charge and achieve an ion concentration of 0.15 M) and OPC water [23] were added. The gradient descent method was used for the energy minimization step. The equilibration step was performed within the canonical NPT ensemble and consisted of five steps involving progressively decreasing positional constraints on the protein atoms. The trajectory length of the first stage was 100 ps, the position constraints were 500 kJ/mol/Å² and the integration step was 0.5 fs. All subsequent stages were 200 ps long with position constraints of 50; 5; 0.5 kJ/mol/Å². The final equilibration step has no constraints. A V-rescale thermostat [24] was used to maintain the system temperature at 300 K; a Parrinello-Rahman barostat [25] was used to maintain the pressure at 1 bar. MD production runs were carried out with an integration step of 2 fs and a trajectory frame recording frequency of 1 ns. The obtained times of the molecular dynamics trajectories were 500 ns. MD trajectories are available for Interactive preview at the following link: https://intbio.org/2024_Druzhkova_et_al/.

Trajectories were processed and aligned using Gromacs 2021.4 [19] and MDAnalysis [18]. RMSF calculations and hydrogen bond analysis were also performed using MDAnalysis. Throughout this paper, the term ‘contact’ is used to refer to a hydrogen bond. A stable interaction was defined as a residue-residue pair that remained interacting in 80% of the MD trajectory frames.

**Docking.** To obtain complexes of SRH and iRGD peptides with their target receptors, docking was performed using the GalaxyPepDock server [26]. Its sampling algorithm includes template selection based on structure and interaction similarity from the database of experimentally determined structures with further building models using energy-based optimization. 10 structures with the best scores are selected and then refined according to the GalaxyRefine protocol. For the submission, the FASTA string of the peptide and the PDB file of the experimentally determined structure of the receptor were used; hydrogens were preliminarily added to the PDB structures using Reduce. Docking was carried out for 3 complexes: SRH peptide with VEGFR2 (template PDB ID 2 × 1 W), iRGD peptide with NRP (template PDB ID 2QQM), and iRGD peptide with integrin α_v_ꞵ_3_ (template PDB ID 1L5G).

FoldX [16] was used to optimize the geometry of each model and to calculate the interaction free energies for 10 models of each complex. In addition, alanine screening (FoldX implementation) was performed for all systems (3). The results of the alanine screening have been summed up by residue along 10 models. The resulting complexes were further estimated by the interaction energy using the MMPBSA method (gmx_MMPBSA [27] implementation). For the MMPBSA analysis, short MD simulation runs of 50 ns were performed for each of the 30 models (10 top conformations of each complex). Only the last 5 ns of the MD trajectories were used for analysis.

### Materials and reagents

Chemicals and reagents were obtained from Applichem (Darmstadt, Germany) unless otherwise specified. Isopropyl-β-d-1-thiogalactopyranoside (IPTG), ampicillin sodium salt, bovine serum albumin (BSA), endothelial cell growth supplement (ECGS) and RIPA buffer were from Sigma-Aldrich (St. Louis, MO, USA). Gibco Bacto tryptone and yeast extract were from Thermo Fisher Scientific, Waltham, MA, USA. Dulbecco’s modified Eagle’s medium (DMEM) and DMEM/F12, glutamine, trypsin-EDTA solution, penicillin, streptomycin and phosphate-buffered saline (PBS) tablets were from PanEco (Moscow, Russia). Fetal bovine serum (FBS) was from HyClone (Cramlington, UK). Endothelial cell growth supplement (ECGS) was from Cell Applications (San Diego, CA, USA). RhVEGF-A165 (Cat. No. PSG010) was from Sci-Store (Moscow, Russia). Cell Counting Kit-8 (CCK-8) reagent was from TargetMol (Japan). Isotonic (0.9%) sodium chloride solution was from Solopharm (St. Petersburg, Russia).

DR5-B and SRH-DR5-B-iRGD proteins were expressed in soluble cell fraction of *E. coli* strain SHuffle B T7 (New England Biolabs, USA) by IPTG induction, and purified by consecutive metal-affinity and ion-exchange chromatography, as described previously [9].

### Cell viability

Human glioblastoma U87MG (cat. no. HTB-14, ATCC, USA) and pancreatic cancer MIA PaCa-2 (Collection of Vertebrate Cell Cultures of the Institute of Cytology RAS, St Petersburg, Russia) cell lines were cultured in DMEM with 10% FBS, 2 mM glutamine (PanEco, Russia), 10 mg/mL penicillin, 10 mg/ml streptomycin at 37 °C, 5% CO_2_. Human umbilical vein endothelial cells HUVEC (Cell Culture Collection of of N.K. Koltzov Institute of Developmental Biology RAS, Moscow, Russia) were cultured in DMEM/F12 with 10% FBS and ECGS. Cell lines were validated by short tandem repeat profiling using COrDIS Plus test system (GORDIZ, Russia). Cells were detached with 0.25% trypsin–EDTA. For viability assay, cells were seeded in 96-well plates at 1 × 10^4^ cell/well. After cell adhesion, DR5-B and SRH-DR5-B-iRGD proteins were added at indicated concentrations for 48 h. 1/10 volume of CCK-8 reagent (TargetMol, Japan) was added for 2 h at 37 °C. Optical density was measured by iMark microplate absorbance reader (Bio-Rad, USA) at 450 nm with background at 655 nm.

For competitive study with VEGFA165, HUVECs were starved in ECGS-depleted DMEM/F12 with 1% FBS for 24 h. Further, cells were incubated with 5 nM or 50 nM DR5-B or SRH-DR5-B-iRGD for 30 min, and 10 ng/ml rhVEGF-A165 was added for 48 h. Cell viability was estimated by CCK-8 as % to control: (OD sample– OD background)/(OD control– OD background) × 100%.

### Flow cytometry

For analysis of DR5, VEGFR2, NRP1 and integrin α_v_β_3_ surface expression, U87MG and MIA PaCa-2 cells were seeded in 25 cm^2^ cell culture flasks (3 × 10^5^ cells/flask) and cultured at 37 °C, 5% CO_2_ for 72 h. Cells were detached with Versene solution, washed with ice-cold PBS and resuspended in FACS buffer (1% BSA in PBS), which was used for all subsequent washing procedures. After washing twice, the cells were fixed with 4% paraformaldehyde for 20 min at room temperature with gentle rotation (Biosan Multi-Bio RS-24, Latvia), washed, and incubated with the appropriate monoclonal antibodies (5 µg/ml) to DR5 (GeneTex, clone DR5-01-1), VEGFR2 (GeneTex, clone 4B3), NRP1 (Invitrogen, clone 5B1E11) or integrin α_v_β_3_ (GeneTex, clone 23C6) for 1 h at 4 °C. The cells were then washed three times and incubated with secondary antibodies Dylight 488 (GeneTex, 20 µg/ml) for 1 h, washed and suspended in FACS buffer. Surface expression of receptors was determined using a CytoFlex flow cytometer (Beckman Coulter, Brea, IN, USA) using mouse IgG1 as an isotype control (Cloud-Clone corp, MAA074Hu22).

### Xenograft studies

Specific pathogene-free (SPF) NU-A/ATyrc/Tyrc Foxn1nu/Foxn1nu, female, 9–13-week-old mice were from Puschino Animal Breeding Facility, branch of the Shemyakin-Ovchinnikov Institute of Bioorganic Chemistry RAS, accredited by Association for Assessment and Accreditation of Laboratory Animal Care. The mice were admitted with a quality certificate and veterinary passport. The animal care was performed according to standard operational procedures.

U87MG or MIA PaCa-2 cells were inoculated subcutaneously at 1 × 10^7^ cells per mouse in Matrigengel Matrix (ABW Mogengel Bio, China) in right dorsal flank. Treatment started after tumors reached 150 ± 20 mm^3^. The mice were divided into groups (*n* = 6) randomly, ligands (20 mg/kg) or vehicle (0.9% sodium chloride, Solopharm, Russia) were administered intravenously in the tail vein 7 times (for U87MG) or 10 times (for MIA PaCa-2) every other day. Animal weight and condition were monitored during all the observation time. Tumor sizes were measured twice a week percutaneously by an electronic digital caliper STORMTM 3C301 «Central». Tumor volumes were calculated by formula V = π/6 × width^2^ × length. Tumor growth inhibition rates were calculated by formula [(Vc × Vex)/Vc] × 100% (Vex, Vc– average tumor volumes in experimental and control groups). At the end of experiments animals were anesthetized intramuscularly with a mixture of Zoletil (40 mg/kg, 50 µL, Virbac SA, France) and 2% Rometar (10 mg/kg, 10 µL, Spofa, Czech Republic) and euthanized by cervical dislocation.

### Optoacoustic microangiography

**Optoacoustic imaging system.** The OA images were obtained on the 30th (for U87MG) or 43rd (for MIA PaCa-2) day of tumor growth using a raster-scan system employing fish-eye detector sensitive to torturous blood vessels of experimental neoplasms [28]. The OA system employed a 532 nm diode-pumped laser (ONDA532, Bright Solutions, Italy) equipped with 400 μm-diameter multimode fiber, a 16-bit 500 MHz analog-to-digital converter (CSE25216, GaGe, USA), and two orthogonally placed V-408.132020 piezo scanners (PI micos, Germany). A spherically focused fish-eye ultrasonic antenna was fabricated based on 25 μm PVDF piezoelectric film, providing a wide detection bandwidth from 0.1 up to 100 MHz [29] and high sensitivity [30].

During the experiment, pre-anesthetized animals were placed on a plastic substrate with an aperture for the tumor. The substrate was attached to the upper edge of an immersion chamber filled with distilled water, which provided acoustic and optical coupling during the scanning. The scanned area was 10 × 10 mm^2^ with a step of 20 μm. The duration of each scan was estimated at approximately 5 min. The effective visualization depth of about 2 mm was chiefly limited by the laser light penetration at 532 nm wavelength.

**Processing of optoacoustic data.** Each set of 3D data was processed using Matlab-based routines, which included bandpass filtering in the range of 7–100 MHz [31] followed by 2D delay-and-sum reconstruction in the frequency domain in the XZ and YZ planes [32]. Also, an algorithm for eliminating motion artifacts based on the autocorrelation of adjacent B-scans (XZ planes) was applied between reconstructions in two orthogonal planes. The reconstructed 3D OA images were further processed for vessel enhancement and quantitative assessment in Avizo Software 2022.2 (Thermo Fisher Scientific, USA) using a customizable workflow with Python instructions for batch 3D processing [33]. After extracting the region of interest within the tumor volume, a series of filters were applied to the OA data, including 3D adaptive histogram equalization and a Structure Enhancement filter for rod-shaped structures with the Hessian Tensor type.

### Optical coherence tomography angiography (OCTA)

OCTA imaging was performed on a homebuilt multimodal OCT system (Institute of Applied Physics RAS, Nizhny Novgorod, Russia) with the following parameters: 1310 nm central wavelength, 15 mW output power, ~ 15 μm spatial resolution, 1.25 mm scanning depth, and 20,000 A-scans per second imaging speed [34]. Vessel imaging was based on the speckle time variation rate of the full complex signal with high-pass filtering of B-scans consisting of highly overlapping A-scans [35]. The complexity of signal computation was reduced by the optically linearized spectrometer, allowing visualization of angiographic images in real time mode. By displaying 3D images as 2D maximum intensity projections we were able to visualize the vascular network *en face* throughout the entire imaging depth [36]. The density of perfused vessels was calculated as the number of pixels of all vessel skeletons in the analyzed area divided by the total number of pixels in this area, based on the OCTA data [37]. The images were obtained on the 34th (for U87MG) or 47th (for MIA PaCa-2) day of tumor growth, after removing a skin flap from the tumor surface, so that the OCTA view field included only tumor vessels.

### Immunohistochemical staining

Tumor nodes were obtained at autopsy on the 34th (for U87MG) or 47th (for MIA PaCa-2) day of tumor growth, fixed in zinc salt-based fixation during 48 hours, embedded in paraffin, and dissected to 4 µm paraffin sections by rotary microtome Leica RM 2125 RTS (Leica, Germany). Paraffin sections were deparaffinized, then blocked with a solution of 3% BSA for 1 hour at room temperature and 3% H_2_O_2_ for 15 min at room temperature, then antibodies to the endothelial marker CD31 (1:500, clone MEC 13.3 (RUO), BD Pharmigen), Ki-67 (1:700, GB111499, ServiceBio) or cleaved caspase-3 (1:500, GB11532, ServiceBio) were added and incubated overnight at 4ºC. The next day, primary antibodies were detected with Goat anti-rabbit IgG polymer kit ImmPress HRP (MP-7451, Vector Laboratories, Inc., USA) and hematoxylin were applied according to the manufacturer’ protocol. Microvessel density was assessed by counting the microvessel profiles stained with antibodies to CD31 on a cut-off area of 0.1 mm^2^ across at least four randomly selected hotspot zones of tumor parenchyma using a fluorescence microscope (BX 51, Olympus, Japan). Histological samples stained with antibodies to Ki-67 or cleaved caspase-3 were examined using the EVOS M7000 Imaging System (Thermo Fisher Scientific Inc., Waltham, MA USA) in transmitted light. The images were collected as objective-calibrated TIFFs and subsequently analyzed and processed in ImageJ/FIJI (National Institutes of Health, Bethesda, Maryland, USA). The plugin for color deconvolution in the H&DAB mode was used for the detection DAB-positive staining and the tool “Analyze particles” was applied for positive cells calculation. Five randomly selected fields of view were analyzed for each slide. The data are presented as Mean ± SD.

### ELISA

Recombinant extracellular domain of DR5 receptor (R&D Systems Inc., USA) was immobilized on ELISA plates overnight at 4 °C at a concentration of 0.5 µg/ml in 100 mM carbonate buffer pH 9.4. Plates were washed three times with PBST (PBS + 0.05% Tween), and wells were blocked with 2% BSA in PBST for 1 h at room temperature. Serum samples (in triplicate) or recombinant DR5-B and SRH-DR5-B-iRGD standards were added at concentrations from 3.9 to 2000 ng/ml and incubated for 1 h at 37 °C. The captured ligands were detected by subsequent incubation with monoclonal antibodies to TRAIL (RnD Systems, MAB375) and mouse IgG (HAF007, R&D Systems, USA) conjugated with horseradish peroxidase. Unbound antibodies were washed three times with PBST and color was developed using OPD colorimetric substrate. Optical density was determined at 490 nm using an iMark spectrophotometer (Bio-Rad, USA). DR5-B concentrations in samples were extrapolated from a four-parameter fit to a DR5-B standard curve.

### Mouse pharmacokinetics

ВALB/c female mice from Puschino Animal Breeding Facility, branch of the Shemyakin-Ovchinnikov Institute of Bioorganic Chemistry RAS, were used for pharmacokinetic studies (*n* = 3 per time point). DR5-B and SRH-DR5-B-iRGD were administered intravenously via the tail as a single bolus at dose 20 mg/kg. Between 3 min and 4 h after the injection, blood samples (300–400 µl) were collected from the inferior vena cava at sacrifice. Blood was allowed to clot at room temperature, and the serum was harvested after centrifugation at 3500 x g for 10 min. Samples were stored at − 70 °C for further analysis of drugs concentrations by ELISA.

### Non-compartmental pharmacokinetics analysis

Pharmacokinetic parameters of DR5-B, including area under the plasma concentration-time curve (AUC), apparent total volume of distribution (Vss), clearance (CL) and serum half-life (t1/2) were estimated by standard non-compartmental methods. The area under the first moment curve (AUMC) was calculated by the trapezoidal rule. The mean residence time (MRT) was calculated as AUMC0-∞/AUC0-∞. Calculation of pharmacokinetic parameters was performed by Microsoft Excel (version 16.3) and GraphPad Prism (version 10.0).

### Western blotting

U87MG and MIA PaCa-2 cells were seeded in 6-well plates at 1.5 × 10^6^ cells/well. After treatment with 50 nM of DR5-B or SRH-DR5-B-iRGD for 4 h, cells were washed with PBS, detached by 2 mM EDTA, centrifuged at 500 g for 5 min, resuspended in RIPA buffer for 30 min on ice and centrifuged at 16,000 g for 15 min. For accumulation studies, the kidney and liver of BALB/c female mice (*n* = 3 per group) were excised, homogenized in RIPA buffer and clarified by centrifugation. Protein concentrations in supernatants were measured with Micro BCA Protein Assay Kit (Thermo Fisher Scientific, USA). Samples were separated by SDS-PAGE (20 µg protein per band) and transferred onto PVDF membranes (Life Technologies, USA). TBST buffer (50 mM Tris-HCl, 150 mM NaCl, 0.05% Tween-20) with 5% nonfat dry milk was used for blocking. The membrane was washed with TBST and incubated with primary antibodies to caspase-8 (Enzo Life Sciences, clone 5F7), caspase-3 (GeneTex, Cat. No. GTX110543), PARP (Invitrogen, clone 123), TRAIL (PeproTech, RRID AB_3074165) or GAPDH (Thermo Fisher Scientific, clone GA1R) in TBST/milk solution overnight at + 4 °C. followed by HRP-conjugated secondary antibodies (BioRad, USA) (1:1000) for 1 h. After washing with TBST, the blots were developed by Clarity Western ECL Substrate (BioRad, USA) and visualized on C-DiGit Blot Scanner (LI-COR Biosciences, USA).

### Statistical analysis

Statistical analysis was performed using GraphPad Prism 8.0.1 (GraphPad Software Inc., San Diego, CA, USA). In cell viability assay, the data represented means ± SD of triplicate assays. The experiments were repeated at least three times with statistical analysis performed using either Student’s *t*-test or two-way ANOVA, as indicated in corresponding sections. In xenograft studies, the significance between groups was tested using two-way ANOVA with Dunnett’s multiple comparisons test. In OA, OCTA, and immunohistochemical studies, Mann-Whitney test or unpaired *t*-test was used, as indicated in corresponding sections. The normality of data distribution was checked by Shapiro-Wilk and Kolmogorov-Smirnov tests. Differences were considered significant at *p* ≤ 0.05.

## Results

### Molecular dynamics simulation of SRH-DR5-B-iRGD fusion protein and its interactions with DR5

Genetic fusion of the DR5-specific TRAIL variant DR5-B with the SRH and iRGD peptides was initially aimed at increasing the specific activity by engaging additional tumor targets. Earlier, we demonstrated that SRH-DR5-B-iRGD binds specifically to DR5, VEGFR2, and integrin α_v_β_3_ receptors with an equilibrium dissociation constants (*K*d) in nanomolar ranges [9]. Here, we have applied molecular modelling to investigate the stability of trimeric SRH-DR5-B-iRGD fusion and its interaction with the target receptors. Molecular simulation of SRH-DR5-B-iRGD binding to DR5 receptor demonstrated that both SRH and iRGD peptide moieties exhibit no interference, and even improve the interaction with the DR5.

**Fig. 1:**
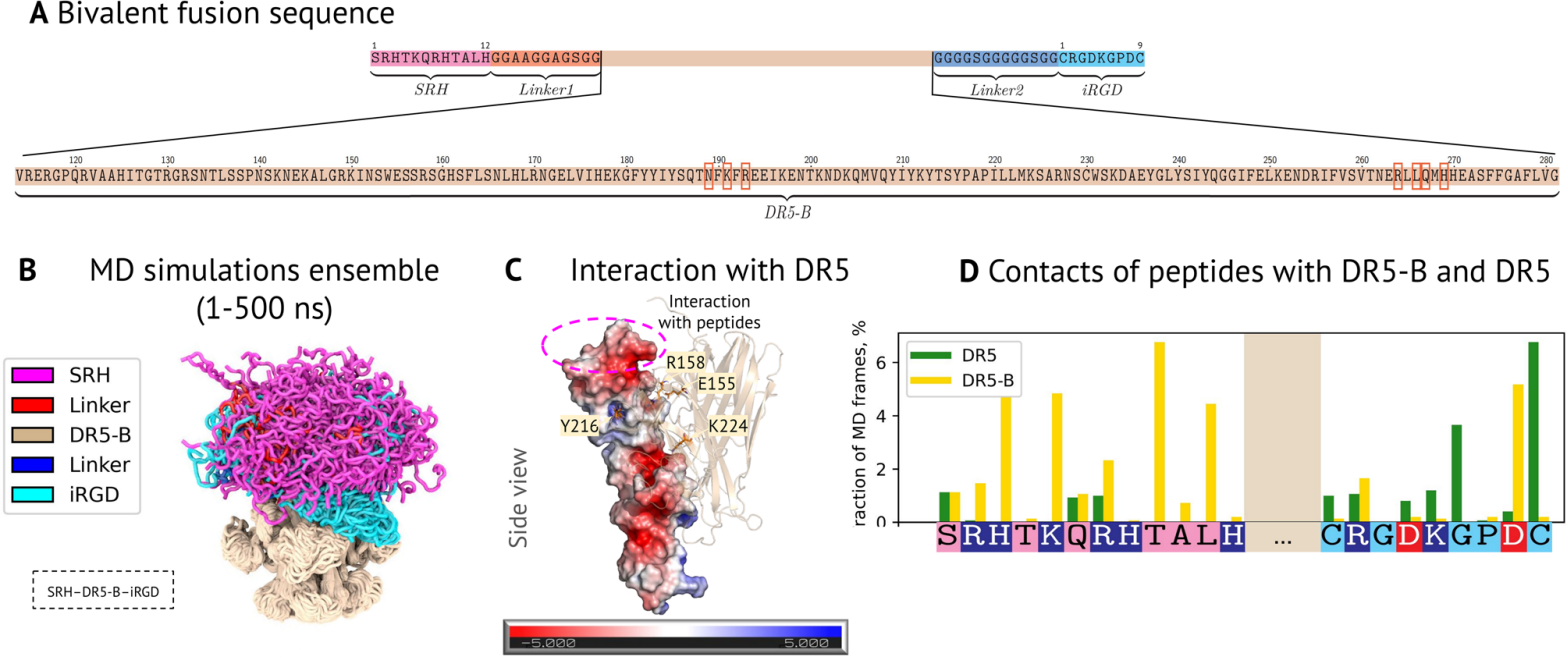
Molecular details of the dynamics of SRH-DR5-B-iRGD and its interactions with DR5. **(A)** SRH-DR5-B-iRGD sequence. Red frames mark amino acid substitutions in TRAIL (DR5-B) sequence for specific DR5 binding **(B)** Overview of the MD simulation trajectory of SRH-DR5-B-iRGD is shown as an overlay of the MD frames. **(C)** Interaction interface between DR5-B and DR5. DR5-B residues that formed stable contacts with DR5 are shown as sticks. DR5 is shown as an electrostatic surface (calculated using the APBS plugin for PyMol). **(D)** Number of contacts formed by peptides with DR5 and DR5-B in the MD simulation. Three dots indicate DR5-B. Uncharged residues of SRH peptide are in pink, and the residues of iRGD peptide are in cyan. Positively and negatively charged residues are in blue and red, respectively

To study the dynamic properties of SRH-DR5-B-iRGD (Fig. 1A) and its interaction with the DR5 receptor in atomistic detail, we performed an all-atom molecular dynamics simulation of the SRH-DR5-B-iRGD trimer with and without DR5 receptor (SRH-DR5-B-iRGD/DR5 and SRH-DR5-B-iRGD, respectively) (Fig. 1B, Fig. S1A, B Interactive trajectory preview at link https://intbio.org/2024_Druzhkova_et_al/). The initial structure was derived from the X-ray structure of trimeric TRAIL with DR5 (PDB ID 1D0G), modified by *C*- and *N*-end fusion with SRH and iRGD peptides and their linkers, correspondingly, and six amino acid substitutions in TRAIL sequence for specific DR5 binding (DR5-B domain) [7] (Fig. 1A).

During the simulation, the globular DR5-B trimeric domain remained stable, while the linkers and peptides retained their disordered structure (Fig. S1A, B). The SRH-DR5-B-iRGD formed stable contacts with the DR5 receptor, all of which involved DR5-B domain residues, but not linker or peptide residues. The most stable interaction was with the R segment, with stable contacts formed by R62, D49, E98 and T52 of DR5 and E155, R158, K224 and Y216 of DR5-B. Interactions with other two receptor segments were more dynamic (Fig. 1C**)**.

Six amino acid substitutions in DR5-B with respect to the TRAIL structure should not complicate the interaction with DR5 according to our MD data. Most of them (Q193R, H264R, I266L, D267Q, D269H) were not involved in the stable DR5-B/DR5 contacts in the MD simulations and the TRAIL-DR5 interface in the X-ray structure (PDB ID 1D0G), confirming that these substitutions are primarily aimed at reducing the interaction of TRAIL with DR4, DcR1, DcR2 and OPG, without affecting the interaction with DR5, supporting previous data [7]. On the other hand, simultaneous substitutions of Y189N and D267Q of DR5-B formed an intra-DR5-B interaction in the MD simulation and presumably stabilized the DR5-B structure.

Surprisingly, SRH and iRGD peptides and their linkers also interacted with DR5, but these interactions were transient, i.e. no stable contacts between peptides and DR5. The DR5 residues involved in transient interactions with overall positively charged SRH and iRGD peptides were localized in the *N*-terminal region of DR5 having negatively charged residues (Fig. 1C). SRH and iRGD formed an average of 0.1 and 0.5 direct contacts with DR5 in the MD simulations, respectively, mainly with the R segment, which maintained more stable contacts with DR5-B. Peptide’ residues, especially SRH residues, also interacted with the DR5-B domain (Fig. 1D).

To further assess whether peptides could weaken interactions between DR5-B and DR5, we performed an additional MD simulation of DR5-B with DR5 without peptides and linkers. Surprisingly, simulation of the system without peptides led to fast dissociation of DR5 from DR5-B (Fig. S2A, B). This was associated with partial disruption of stable DR5-B-DR5 contacts. Dissociation events were observed for two of the three receptor chains: S and T, while chain R remained in contact with DR5-B. Similar dissociation with lower amplitude was present in the simulation with peptides for segment S (Fig. S2C), which did not form contacts with peptides.

Thereby, MD simulations showed that the engineered fusion SHR-DR5-B-iRGD has a stable globular domain structure with disordered peptides and linkers. The peptides do not compete with DR5 in interaction with DR5-B, and DR5-B-specific substitutions have little effect on them. In addition, MD data suggest that the peptides stabilize the DR5-B-DR5 complex through interaction with DR5.

### Interactions of SRH and iRGD peptides with target receptors

**Fig. 2:**
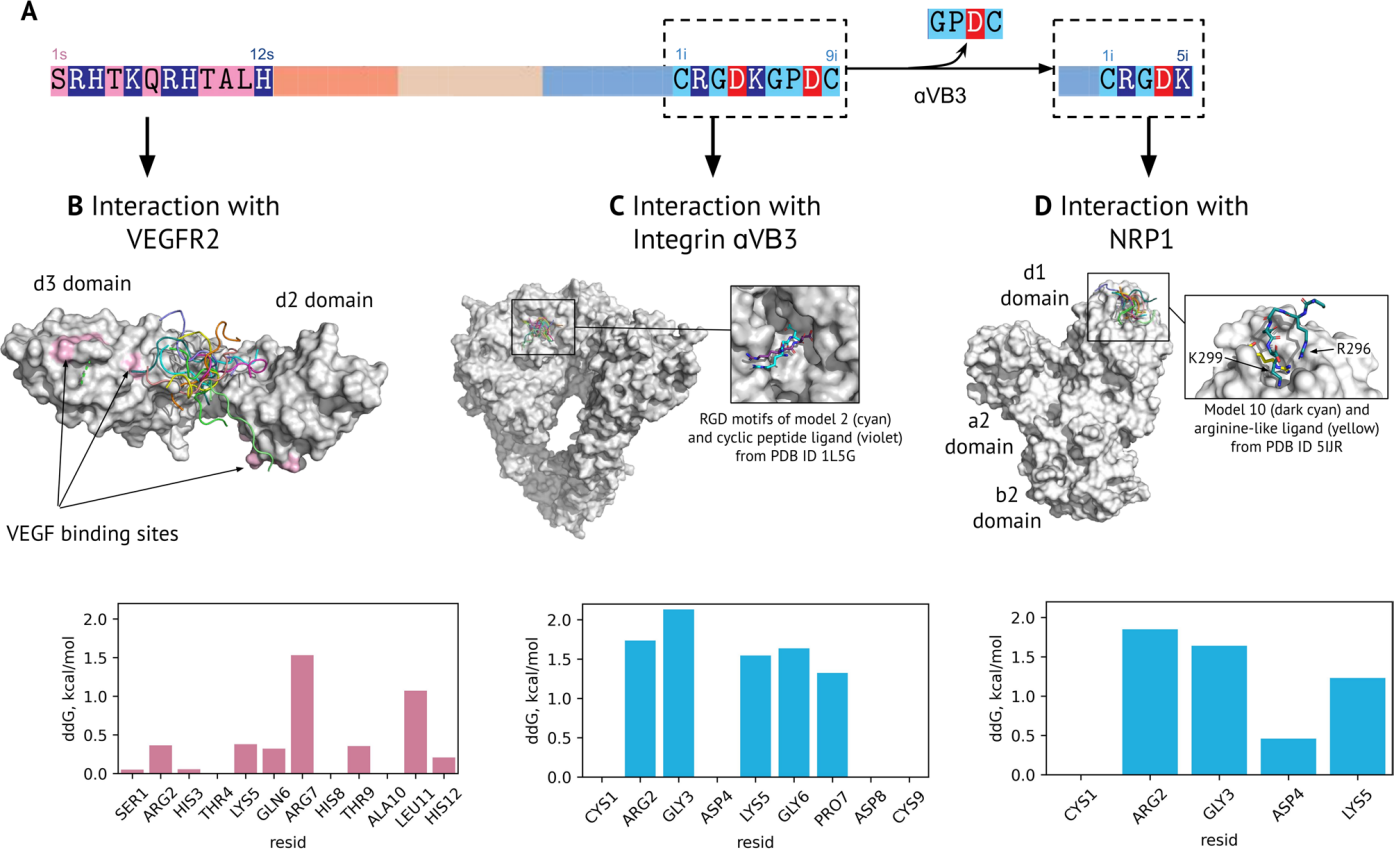
Peptide docking to target receptor domains. (**A**) Schematic representation of SRH-DR5-B-iRGD sequence and iRGD hydrolysis by integrin α_v_β_3_. (**B-D**) Results of peptide docking to its receptors. A set of docking solutions of each complex is shown in the colored cartoon, with the domains of the target receptors shown in the gray area. Representative solutions (with the lowest interaction free energy) are shown in black frames when zoomed in and compared with the experimental structure of the ligand in the binding sites. The histograms below show the result of the alanine screening of the docking solutions, summed for each peptide residue along models

The next step was to characterize structurally the peptides that bind to their target receptors. Three complexes were docked: SRH with VEGFR2, unmodified iRGD with integrin ɑ_v_ꞵ_3,_ and hydrolyzed iRGD with NRP1 (Fig. 2A, interactive materials at link https://intbio.org/2024_Druzhkova_et_al/). SRH was derived from the peptide designed to bind the d2-d3 domains of VEGFR2 [38]. In our results, SRH interacted with the hinge region between the d2 and d3 domains close to the VEGFA binding sites [39], but did not bind them accurately (Fig. 2B). The largest contribution to the interaction free energy was provided by Arg 7(s). Weaker yet important interactions were provided by Arg 2(s), Lys 5(s), Gln 6(s), Thr 9(s) and His 12(s), whose contacts were electrostatic and polar in nature. A notable effect was observed for Leu 11(s) contacts formed with the hydrophobic region of VEGFR2 in models 8 and 9.

The integrin α_v_β_3_ has an RGD-binding motif [40], which was also recruited by the RGD motif of the iRGD peptide (Fig. 2C). Some predicted complexes replicated the geometry of the RGD motif in the binding site well enough with respect to known experimental complexes. As the docking software does not support disulfide bonds, a linear peptide structure was generated. Nevertheless, some solutions had cysteine close enough to interact.

The hydrolyzed iRGD interacts with NRP1 in a known binding site for arginine-like ligands (“acidic groove” of the b1 domain [41]). The docking results included two possible modes of iRGD binding: with insertion of Arg 2(i) or C-terminal Lys 5(i) into the binding site. Although the latter is more preferable, the inserted Lys 5(i) had 7 out of 10 docking solutions and the interaction free energy of the best solution with inserted Lys 5(i) was lower than the best solution with inserted Arg 2(i). It should also be noted that the docking was performed with the intact peptide, whereas Arg 2(i) is attached to the DR5-B domain via a linker.

### Assessment of in vitro activity of SRH-DR5-B-iRGD in human glioblastoma U87MG and pancreatic cancer MIA PaCa-2 cells

**Fig. 3:**
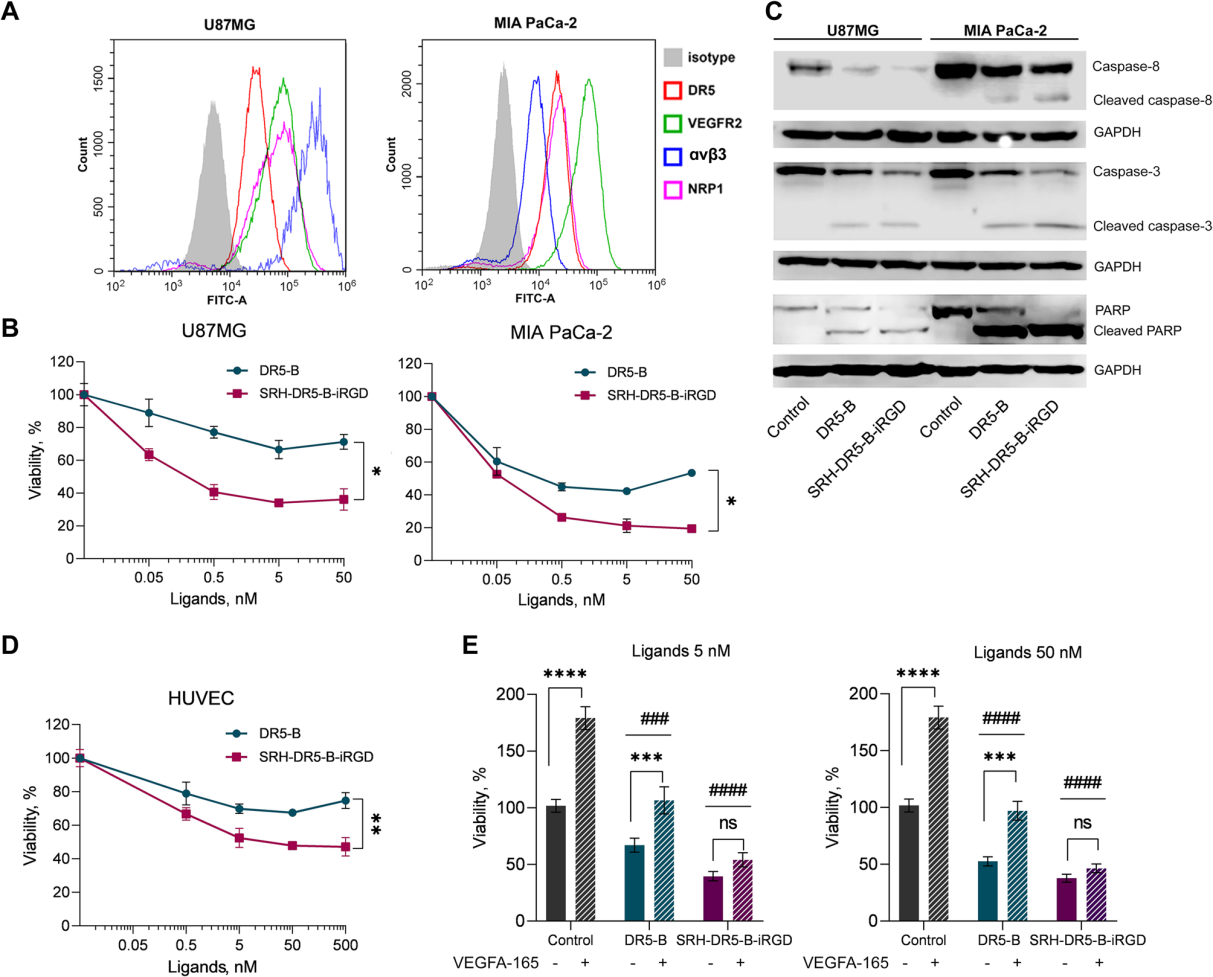
Comparative activity of DR5-B and SRH-DR5-B-iRGD in vitro. (**A**) Expression level of DR5, VEGFR2 and integrin α_v_β_3_ receptors in U87MG and MIA PaCa-2 cell lines, flow cytometry; (**B**) SRH-DR5-B-iRGD exhibits enhanced cytotoxic activity compared to DR5-B in U87MG and MIA PaCa-2 cell lines, Student’s *t*-test, *p < 0.05; (**C**) Caspase-3 and PARP are cleaved more effectively after SRH-DR5-B-iRGD rather than DR5-B treatment; Cells were incubated with 50 nM of ligands for 4 h and analyzed by western blotting; (**D**) Both DR5-B and SRH-DR5-B-iRGD are cytotoxic to HUVEC cells. Student’s *t*-test, ***p < 0.0005; (**E**) SRH-DR5-B-iRGD inhibit VEGFA165-induced HUVEC proliferation more efficiently than DR5-B. Statistical analysis of VEGFA165-treated vs. non-treated groups: ***p < 0.0005, ****p < 0.0001, analysis of ligands-treated vs. control groups: ^###^*p* < 0.0005, ^####^*p* < 0.0001, two-way ANOVA with Sidak ‘s multiple comparisons test

We have previously shown that SRH-DR5-B-iRGD exhibits enhanced cytotoxicity compared to DR5-B in various human tumor cell lines. In particular, glioblastoma U87MG and pancreatic cancer MIA PaCa-2 expressed all three target receptors DR5, VEGFR2 and integrin α_v_β_3_ (Fig. 3A), and were highly sensitive to SRH-DR5-B-iRGD in vitro (Fig. 3B). Caspase-8, caspase-3 and PARP cleavage indicated that SRH-DR5-B-iRGD exhibits its cytotoxic activity by apoptotic mechanism. Importantly, caspase-8, caspase-3 and PARP cleavage was more pronounced after treatment with SRH-DR5-B-iRGD rather than DR5-B (Fig. 3C). Due to high tumorigenicity, these cell lines were chosen for further evaluation of anti-angiogenic properties of SRH-DR5-B-iRGD in vivo.

### SRH-DR5-B-iRGD exhibits cytotoxicity and inhibits VEGFA165-induced proliferation of human umbilical vein endothelial cells (HUVEC) more efficiently than DR5-B

TRAIL was previously reported to inhibit VEGF-induced proliferation of HUVEC in vitro and angiogenesis in vivo [42, 43]. In line with those findings, a dose-dependent decrease in viability was also observed for DR5-specific TRAIL variant DR5-B, yet it was even more pronounced for SRH-DR5-B-iRGD (Fig. 3D). It should be noted that bispecific fusions SRH-DR5-B and DR5-B-iRGD demonstrated the intermediate effect between DR5-B and SRH-DR5-B-iRGD, revealing the impact of each of SRH and iRGD effector peptides within the SRH-DR5-B-iRGD fusion protein (Fig. S3). Stimulation of the cell proliferation by vascular endothelial growth factor VEGFA165 was inhibited by both DR5-B and SRH-DR5-B-iRGD ligands, however the inhibition by SRH-DR5-B-iRGD was stronger (Fig. 3E).

### SRH-DR5-B-iRGD exhibits enhanced antitumor activity over DR5-B in xenograft models of glioblastoma and pancreatic cancer in vivo

**Fig. 4:**
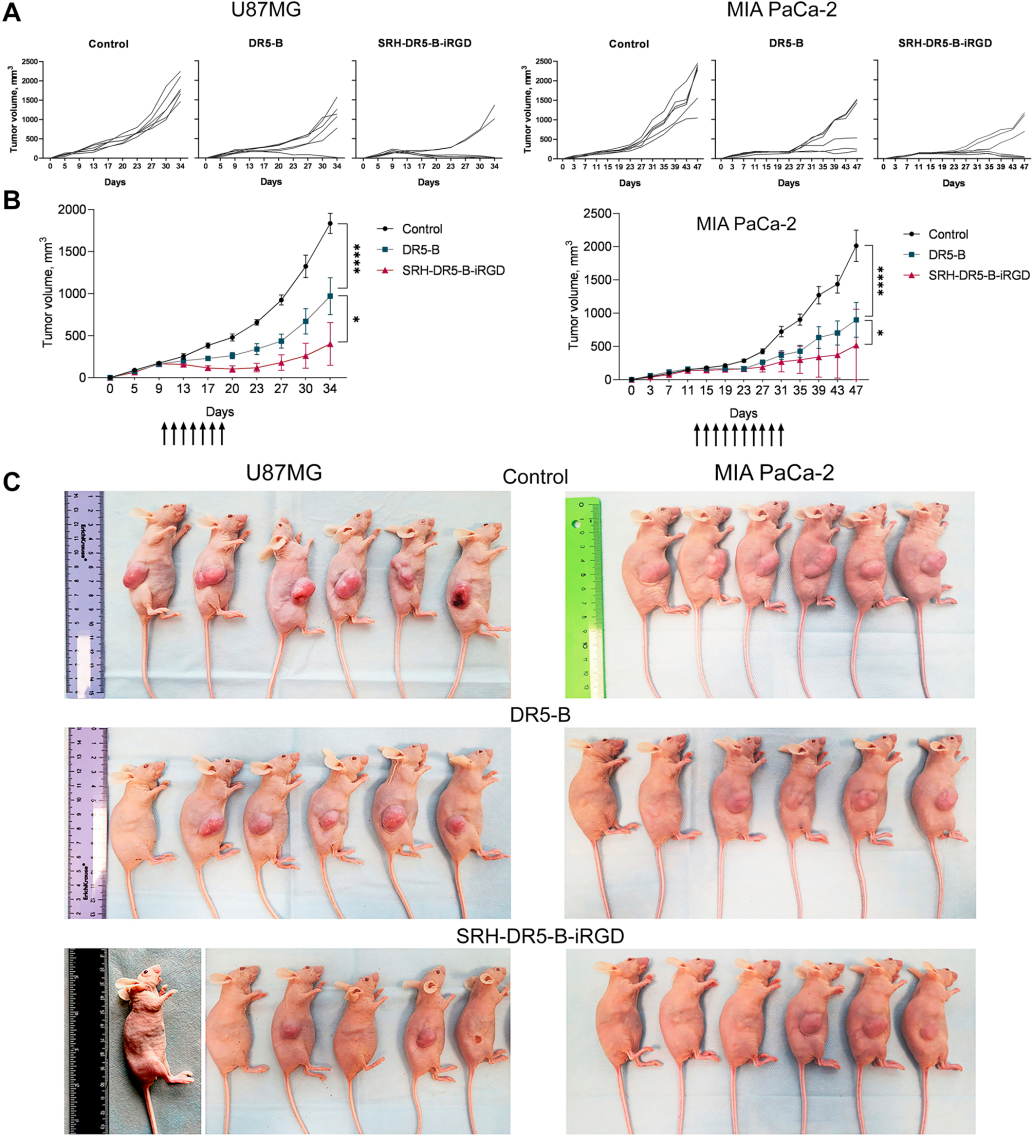
Comparative antitumor activity of 20 mg/kg DR5-B and SRH-DR5-B-iRGD in vivo in human glioblastoma U87MG (left panel) and pancreatic cancer MIA PaCa-2 (right panel) subcutaneous xenograft models in nude mice. (**A**) Tumor volume growth curves of individual mice in each group (*n* = 6); (**B**) Averaged tumor volume growth curves (*n* = 6). Significance between SRH-DR5-B-iRGD vs. DR5-B was analyzed using two-way ANOVA followed by Dunnett’s post hoc test: ***p < 0.0001, **p < 0.005, *p < 0.05; (**C**) Mice with xenografted tumors from the control and treated groups at 34th (U87MG, left panel) or 47th (MIA PaCa-2, right panel) day of tumor growth

The comparative tumor growth suppression by DR5-B and SRH-DR5-iRGD (20 mg/kg) was investigated on the subcutaneous xenograft models of glioblastoma U87MG and pancreatic cancer MIA PaCa-2. In U87MG model, SRH-DR5-B-iRGD induced complete tumor regression in 1 out of 6 mice, and significant regression of 3 out of 6 tumor nodes, whereas DR5-B caused the significant tumor regression in only 1 out of 6 mice. Tumor growth inhibition (TGI) rate in SRH-DR5-B-iRGD group was 74% compared with 45% in DR5-B group. Similar effect was observed in MIA PaCa-2 model: SRH-DR5-B-iRGD induced significant tumor regression in 3 out of 6 mice, whereas DR5-B induced considerable regression in 2 out of 6 animals (Fig. 4A, B). TGI rate by SRH-DR5-B-iRGD was 69% compared with 51% by DR5-B. Importantly, neither DR5-B nor SRH-DR5-B-iRGD have triggered body weight loss or histopathological changes in mice, indicating good safety profile (Fig. S4).

### SRH-DR5-B-iRGD reduces tumor vascularization, as revealed by in vivo OA microangiography

**Fig. 5:**
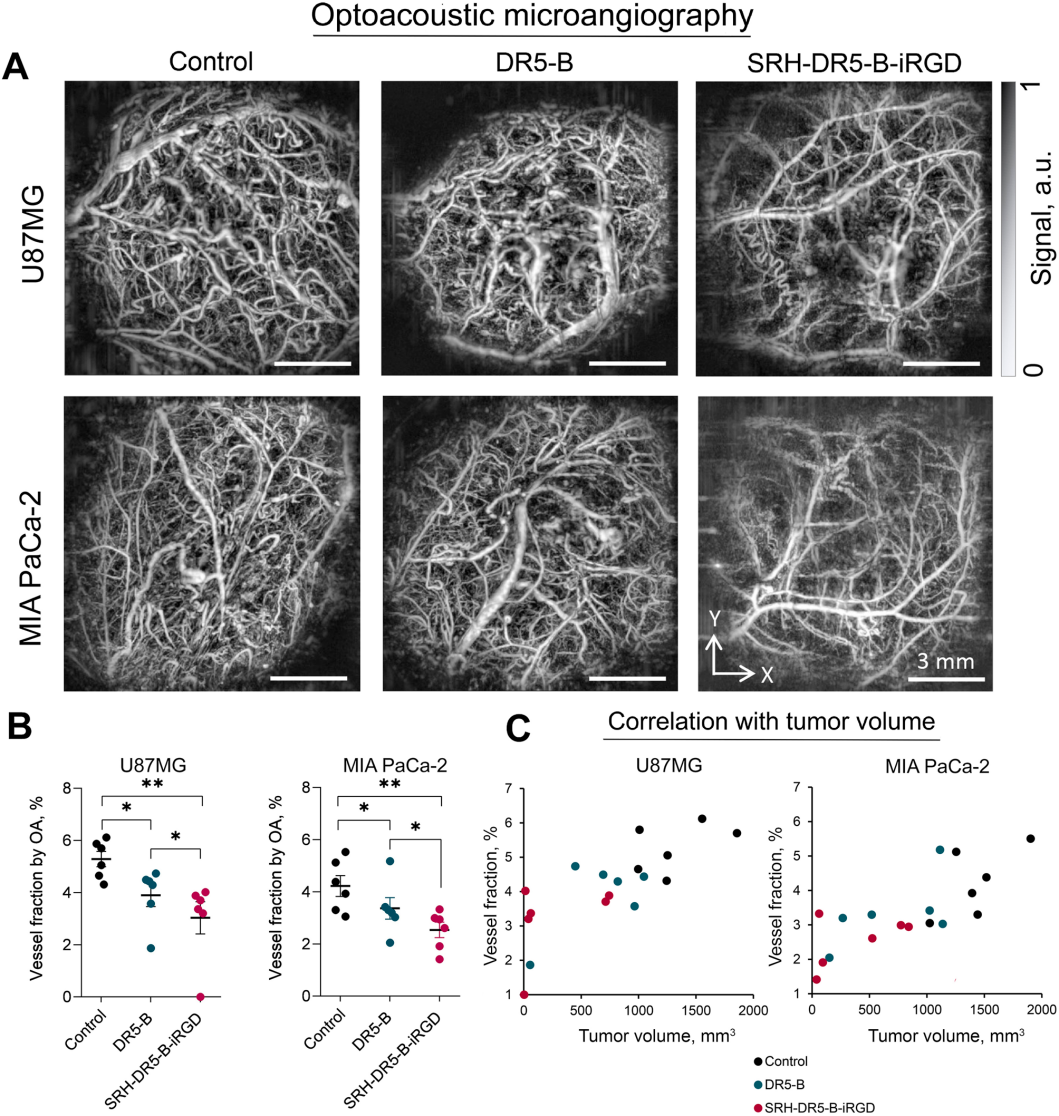
In vivo optoacoustic (OA) microangiography of xenograft U87MG and MIA PaCa-2 tumors treated with DR5-B or SRH-DR5-B-iRGD visualizes tumor microvessels above 30 μm in diameter. (**A**) Examples of OA images of U87MG and MIA PaCa-2 xenograft tumor nodes after treatment with DR5-B and SRH-DR5-B-iRGD; (**B**) Vessel fractions assessed by the OA microangiography, Mann-Whitney test (U87MG) or unpaired *t-*test (MIA PaCa-2), *p < 0.05, **p < 0.01; (**C**) Correlation plots of vessel fraction values with tumor volumes

The tumor vasculatures of DR5-B and SRH-DR5-B-iRGD-treated mice were quantitatively assessed in vivo by OA imaging on the 30th (for U87MG) or 43rd (for MIA PaCa-2) day of tumor growth. Figure 5A presents OA images illustrating the differences in vessel structure between untreated and treated tumors. Control tumors in both U87MG and MIA PaCa-2 models are characterized by a large number of tortuous, unevenly distributed vessels of varying calibers. Slightly higher vascularization was observed in the U87MG model compared to the MIA PaCa-2 model (Fig. 5A), with average values of vessel fractions of 5.2 ± 0.7% and 4.2 ± 1.0%, respectively (Fig. 5B), and nonsignificant differences between models.

Images of DR5-B-treated tumors show a reduction in vascularization, indicating statistically significant effect of the drug on blood vessel formation. The averaged vessel fraction values decreased to 3.9 ± 1.1% in U87MG (*p* = 0.02) and to 3.4 ± 1.0% in MIA PaCa-2 cells (*p* = 0.04) (Fig. 5B). However, SRH-DR5-B-iRGD inhibited blood vessel growth more significantly in both tumor models. OA images revealed a reduction in the number of visualized vessels and the formation of extensive avascular zones within the tumors (Fig. 5A). For U87MG, mean vessel fraction values dropped 1.7 times from the untreated group (*p* = 0.002). For MIA PaCa-2, the vessel fraction decreased 1.6 times (*p* = 0.007) (Fig. 5B).

Analysis of the relationship between vascularization and tumor volume in treated and untreated animals demonstrated that an increase in vessel fraction values is associated with an increase in tumor size (Fig. 5C). A strong positive correlation between these parameters was observed for both the U87MG model (Pearson correlation coefficient *r* = 0.77, *p* < 0.01) and the MIA PaCa-2 model (*r* = 0.78, *p* < 0.01).

### OCTA in vivo confirms OA data on the reduction of tumor vascularization by SRH-DR5-B-iRGD

**Fig. 6:**
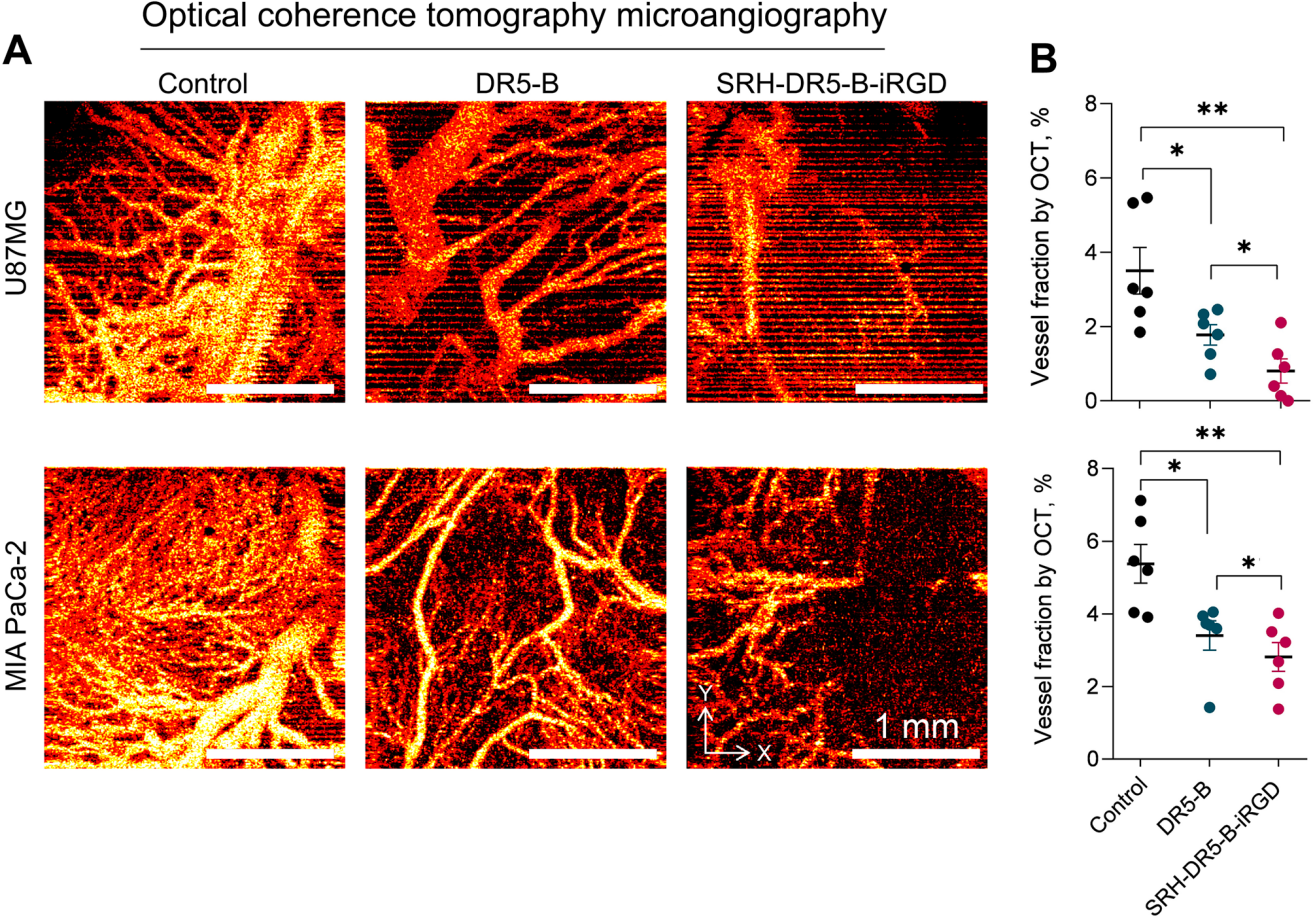
Optical coherence tomography-based angiography (OCTA) imaging of the xenograft U87MG and MIA PaCa-2 tumors treated with DR5-B or SRH-DR5-B-iRGD. (**A**) Representative 2D projections of 3D OCTA images of U87MG and MIA PaCa-2 tumor nodes after DR5-B or SRH-DR5-B-iRGD treatment; (**B**) Vessel fractions assessed by OCTA imaging, unpaired *t-*test (U87MG) or Mann-Whitney test (MIA PaCa-2), *p < 0.05, **p < 0.01

The xenograft tumor nodes were further visualized by optical coherence tomography angiography (OCTA), an imaging technique that allows to quantitatively assess the density of perfused vessels with active blood flow. The OCTA images were obtained on 34th (for U87MG) or 47th (for MIA PaCa-2) day of tumor growth (Fig. 6A).

The density of perfused vessels in the control groups was consistent with the vascular density visualized by OA in both U87MG and MIA PaCa-2 models. MIA PaCa-2 tumor nodes contained slightly more perfused vessels compared with U87MG in all groups (*p* < 0.05, estimated by two-way ANOVA with Sidak’s multiple comparisons test). In congruence with the OA imaging, OCTA showed a reduction in perfused vessel density in DR5-B-treated groups in both models (Fig. 6B). Similarly, in SRH-DR5-B-iRGD-treated groups, reduction in the density of perfused vessels was more pronounced. This may be explained by rapid blood flow impairment accompanying tumor node regression in DR5-B-treated tumors, whereas SRH-DR5-B-iRGD caused not only blood flow disruption, but also small-caliber vessels degradation in the tumors (Fig. 6B).

### Immunohistochemical staining reveals reduction of microvessel density following SRH-DR5-B-iRGD but not DR5-B treatment

The tumor nodes were excised during autopsy on the 34th (for U87MG) or 47th (for MIA PaCa-2) day of tumor growth and stained for endothelial marker CD31. The absolute numbers of microvessel profiles per 1 mm^2^ of the histological sections were established morphometrically. The U87MG tumor nodes of all experimental animals (*n* = 6 per group) were subjected to morphometrical analysis to estimate the statistical significance of microvessel distribution. In MIA PaCa-2 model, 1 tumor node per group was selected for representative analysis.

**Fig. 7:**
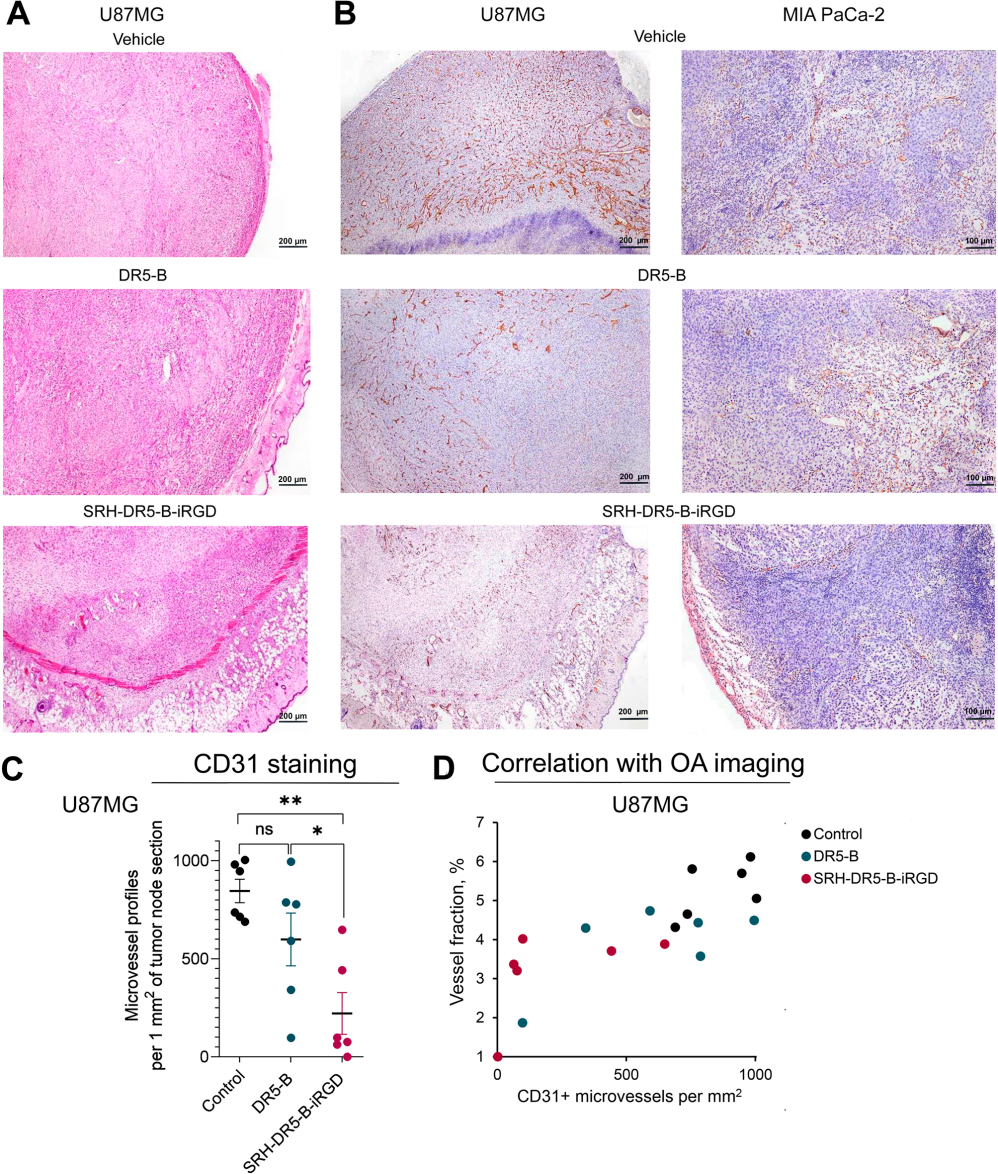
Immunohistochemical analysis of tumor vasculature after treatment with DR5-B or SRH-DR5-B-iRGD. (**A**) Morphological characteristics of U87MG xenografts after treatment with DR5-B or SRH-DR5-B-iRGD, hematoxylin and eosin staining; (**B**) Representative images of U87MG (left panel) and MIA PaCa-2 (right panel) tumor nodes stained for endothelial marker CD31, microvessel profiles are in maroon; (**C**) Morphometric analysis of U87MG microvessel profiles per 1 mm^2^ of the histological sections of tumor nodes (*n* = 6 per group), Mann-Whitney test, *p < 0.05, **p < 0.01; (**D**) Correlation of CD31 + microvessels per mm^2^ with vessel fraction estimated by OA in U87MG model

In control groups of both models, microvessels lacked hierarchical organization and were represented by capillaries with different morphology. In SRH-DR5B-iRGD-treated group of U87MG xenografts, in 1/6 animal with complete tumor regression the mature scar was formed, and tumor cells were not found. In 3/6 mice with significant tumor regression, the remnants of tumor nodes were abundantly infiltrated with macrophages and lymphocytes. The histological preparations from the 2/6 mice with moderate tumor regression contained sections of large tumor nodes with extensive necrosis in the core. Clusters of full-blooded microvessels with sharply dilated lumens were identified in the tumor parenchyma along the periphery of the nodes, indicating altered hemodynamics in tumor microvessels (Fig. 7A).

CD31 immunostaining revealed a decrease in vascularization of viable parenchyma in the SRH-DR5-B-iRGD-treated nodes compared with the untreated group. In DR5-B-treated mice, the morphology of tumor vessels, their distribution and degree of vascularization changed insignificantly compared with the control group (Fig. 7B, C). Similar trends were observed in MIA PaCa-2 representative sections (Fig. 7B, C). Importantly, the number of CD31 + microvessels in U87MG model exhibited a strong positive relationship with vessel fraction values quantified by OA (*r* = 0.76, *p* < 0.01) (Fig. 7D), confirming its utility for characterizing the vascularization of experimental tumors in vivo.

**Fig. 8:**
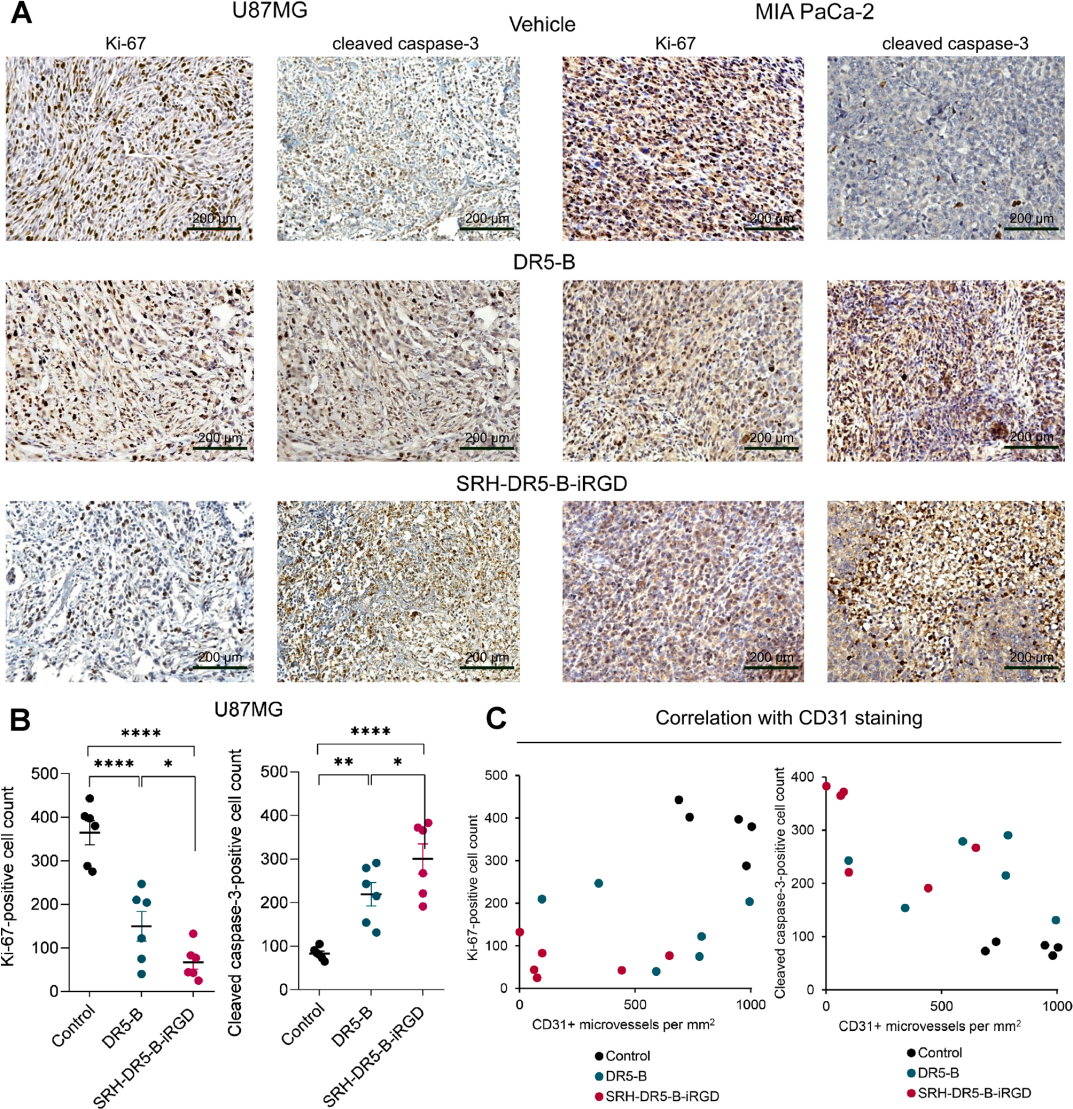
Immunohistochemical staining of tumor nodes for Ki-67 and cleaved caspase-3 after treatment with DR5-B or SRH-DR5-B-iRGD. (**A**) Representative images of U87MG and MIA PaCa-2 tumor nodes stained for Ki-67 and cleaved caspase-3 (in maroon); (**B**) Average number of Ki-67 or cleaved caspase-3-positive cell count per field (×200) of the histological sections of U87MG tumor nodes (*n* = 6 per group), Mann-Whitney test, *p < 0.05, **p < 0.01, ****p < 0.001; (**C**) Correlation of CD31 + microvessels per mm^2^ with Ki-67 or cleaved caspase-3-positive cell count per field (×200) in U87MG model

Additionally, the sections were stained for Ki-67 to identify the proliferating tumor cells, and for cleaved caspase-3, a marker of cell death (Fig. 8). A similar trend was observed as for CD31 staining: a significant decrease in Ki-67 was revealed after both DR5-B and SRH-DR5-B-iRGD treatment in both U87MG and MIA PaCa-2 models. This was accompanied by the increase in cleaved caspase-3 staining. In tumors treated with SRH-DR5-B-iRGD, this effect was pronounced more significantly (Fig. 8A, B). Thereby, the number of CD31 + microvessels positively correlated with Ki-67-positive cell count, and inversely correlated with cleaved caspase-3-positive cell count, as it was estimated in U87MG model (Fig. 8C).

### Pharmacokinetics study of DR5-B and SRH-DR5-B-iRGD

**Fig. 9:**
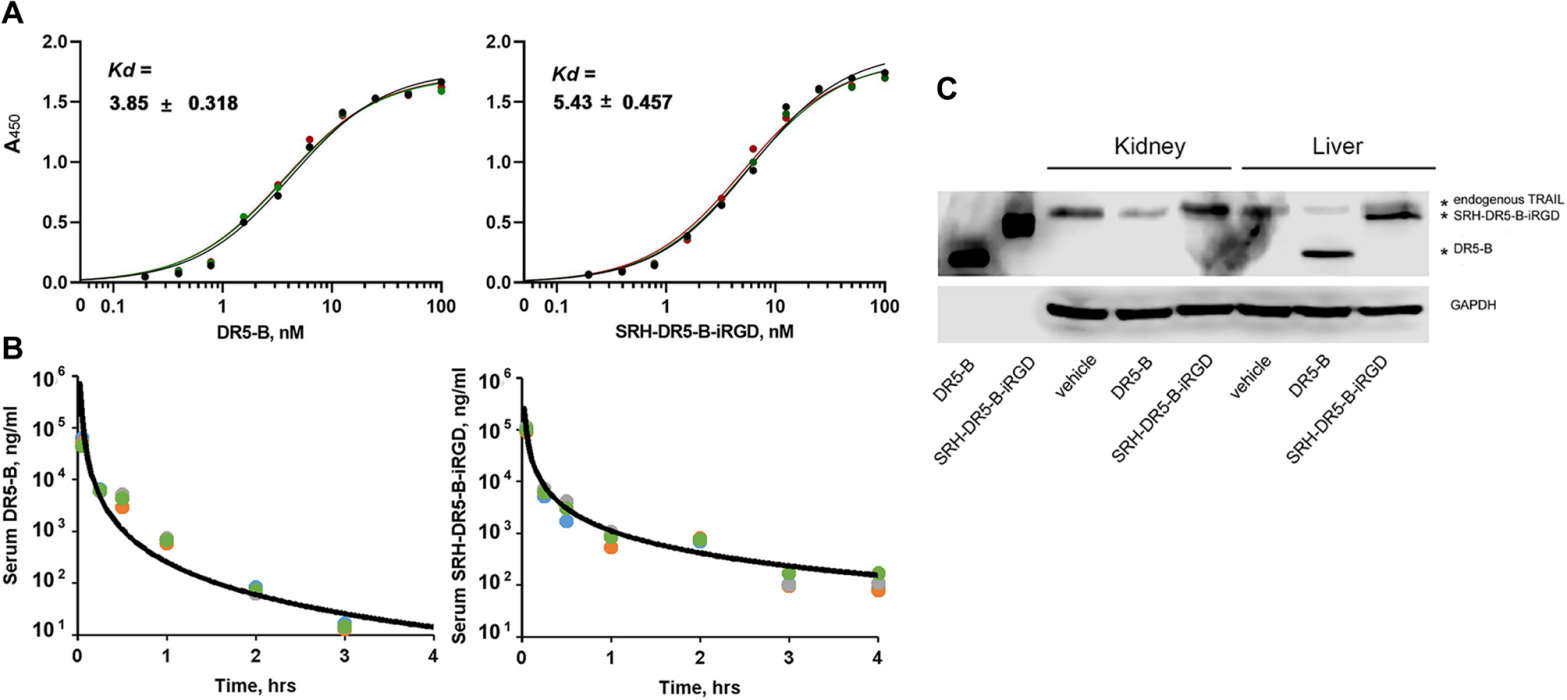
Serum concentrations of DR5-B and SDR-DR5-B-iRGD as a function of time profiles. (**A**) The binding affinity of DR5-B and SRH-DR5-B-iRGD to the DR5 receptor analyzed by ELISA; (**B**) Pharmacokinetic curves of drugs after a single intravenous administration at a 20 mg/kg to BALB/c mice; (**C**) Accumulation of DR5-B and SRH-DR5-B-iRGD (20 mg/kg) in kidneys and liver 4 h after intravenous injection, analyzed by western blot

For pharmacokinetics studies, DR5-B and SRH-DR5-B-iRGD were administered intravenously via the tail vein of BALB/c mice as a single bolus at dose 20 mg/kg. Blood samples (300–400 µl) were collected between 3 min and 4 h after the injection. The concentrations of the drugs in serum were calculated based on the DR5-B and SRH-DR5-B-iRGD enzyme-linked immunosorbent assay (ELISA) titration curves (Fig. 9A). Both ligands had similar affinity for the DR5 receptor (equilibrium dissociation constant *Kd* 3.85 ± 0.318 nM and 5.43 ± 0.457 nM for DR5-B and SRH-DR5-B-iRGD, respectively), as we have shown previously [9]. Sample concentrations were calculated based on the approximated calibration curve obtained from the sandwich ELISA results. A sixth-degree polynomial approximation (R² >0.99) constructed in MS Excel was used to interpolate OD values. The pharmacokinetic curves of both ligands had a distinct biexponential character (Fig. 9B).

The pharmacokinetic parameters of DR5-B and SRH-DR5-B-iRGD studied in mice were comparable (Table 2), however the half-life of SRH-DR5-B-iRGD (42 ± 3.6 min) was 1.4-fold higher compared with DR5-B (30 ± 6.1 min). The rapid elimination of ligands is probably regulated by renal excretion, as can be judged by the pharmacokinetic curves (Fig. 9A) and the rapid clearance. Pharmacokinetic studies of TRAIL in several rodent and primate species suggested renal clearance as the main route of protein elimination [44].

Mean residence time (MRT) was short and varied within eight of minutes range for DR5-B and SRH-DR5-B-iRGD, even *C*_max_ was almost twice higher for SRH-DR5-B-iRGD (Table 2).

**Table 2:** Non-compartmental Pharmacokinetic parameters of DR5-B and SRH-DR5-B-iRGD in mice

Protein	Body Wt.	C_max_	AUC0-∞	t_1/2_	CL	V_ss_
	kg	µg/ml	µg/ml/h	min	ml/kg/h	ml/kg
DR5-B	0.028 ± 0.002	54.2 ± 9.6	10.5 ± 1.4	30 ± 6.1	1905 ± 950	1374 ± 1192
SRH-DR5-B-iRGD	0.028 ± 0.002	103 ± 9.9	17.0 ± 1.9	42 ± 3.6	1175 ± 921	1186 ± 1002

Abbreviations: C_max_, maximum concentration; AUC0-∞, area under the concentration time curve; t_1/2_, terminal half-life; CL, clearance; V_ss_, volume of distribution

Additionally, we have investigated the accumulation of DR5-B and SRH-DR5-B-iRGD in liver and kidneys of BALB/c mice after a single administration. Both DR5-B and SRH-DR5-B-iRGD were not found in kidneys, but were detected in liver in 4 h after intravenous administration at a dose of 20 mg/kg (Fig. 9C). This is supported by the previous data of TRAIL accumulation in liver [45]. Despite the slightly enhanced half-life of SRH-DR5-B-iRGD in plasma, no difference between DR5-B and SRH-DR5-B-iRGD liver accumulation was observed.

## Discussion

TRAIL is a cytokine with pleiotropic effects. In addition to immune surveillance, it plays an important role in endothelial cell physiology. Secchiero et al. reported a proangiogenic role of TRAIL by protecting HUVEC from apoptosis induced by trophic deprivation [46]. In contrast, TRAIL is known to exert antimitogenic effects on HUVEC, as well as inhibited VEGF-induced vessel formation in vitro and in vivo [43]. Cantarella et al. reported the concentration-dependent TRAIL effect in endothelial cells: at low concentrations, TRAIL promoted either mitogenesis or migration of HUVEC, thus promoting angiogenesis, whereas high concentrations induced apoptosis of HUVEC [47]. A very recent study showed that endothelial cell-derived TRAIL stimulates microvessel networks and stabilizes patients with ischemia caused by peripheral artery disease. However, the authors mention that higher levels of TRAIL are usually used for apoptosis induction in cancer cells [48]. We thereby assumed that engaging additional tumor targets to DR5 activation may shift the balance towards anti-angiogenic effect in addition to the main antitumor function of TRAIL.

Vascular endothelial growth factor receptor 2 (VEGFR2) is one of the main targets for anti-angiogenic tumor therapy, as its activation with VEGFA induces proliferation, migration, and angiogenesis in solid tumors [49]. Integrin α_v_β_3_ is also a well-known target for anti-angiogenic cancer therapy due to its high expression on activated endothelial cells and tumor cells, but not on resting endothelium and normal cells. For example, an anti-integrin α_v_β_3_ monoclonal antibody and cyclic peptidic integrin α_v_β_3/5_ antagonist are currently in clinical trials for anti-angiogenic tumor therapy [50]. Thus, the reciprocal functional cross-talk between VEGFR2 and integrin α_v_β_3_ is important for angiogenesis [51].

In order to enhance antitumor efficacy in vivo, we have recently developed the SRH-DR5-B-iRGD protein that affects not only tumor cells, but also the tumor vasculature. The VEGFR2-specific SRH and integrin α_v_β_3_/NRP-1-specific iRGD moieties are both known to exert antiangiogenic functions. For example, in a recent work, iRGD peptide was studied as affinity targeting ligand for treatment of pathological choroidal neovascularization [38, 52]. Molecular modeling showed that genetic fusion of the DR5-specific TRAIL variant DR5-B with SRH and iRGD peptides not only enabled the engagement of additional tumor targets, but also improved the interaction with DR5 receptor. Remarkably, unlike SRH-DR5-B-iRGD, simulation of DR5-B interaction with DR5 was unstable. This may indicate that other agents participate in TRAIL/DR5 interaction. For example, cell surface heparan sulfate was shown to form a ternary complex with TRAIL and DR5, promoting TRAIL-induced apoptosis [53]. Further studies are needed to determine whether this mechanism is essential for DR5 receptor activation upon TRAIL binding and to identify any additional factors involved.

The concept of disrupting tumor angiogenesis by vascular targeting with engineered proteins is supported by other studies. A TAT-AT7 fusion protein designed by attaching the cell-penetrating peptide TAT to a vascular-targeting peptide AT7, competitively bound to VEGFR2 and NRP-1, preventing VEGFA165 binding and targeting glioma neovascularization in vivo [54]. Another VEGFR-targeting fusion protein composed of VEGFR1 peptide antagonist and elastin-based polypeptide inhibited VEGFA-triggered angiogenesis in vitro, and suppressed choroidal neovascularization in a wet age-related macular degeneration mouse model in vivo [55].

To date, OA imaging has proven its applicability for studying the effects of clinically approved antiangiogenic drugs on the vasculature of experimental tumors. In particular, reduction of tumor vessel density [56] and a decrease of hemoglobin content [57], were revealed by OA under action of trebananib and bevacizumab, respectively. OA can also be used to study the response of tumor vessels to new experimental drugs [58]. The OCTA technique additionally provides a map of perfused vessels, which can reveal local microcirculation alterations [59]. Our work is the first to provide a direct in vivo comparison of the antiangiogenic effects of experimental drugs DR5-B and SRH-DR5-B-iRGD by OA and OCTA, which was further confirmed by standard ex vivo method for assessing the number of microvessels.

Importantly, the anti-angiogenic properties of SRH-DR5-B-iRGD were consistent in two different xenograft models. This indicates its potential versatility and suitability for treatment of various types of vascularized solid cancers.

One potential limitation is the limited spatial resolution of the in vivo imaging methods used in the current study [28]. While both OA and OCTA could reliably detect 30 μm in diameter or larger vessels, the average size of capillaries is smaller (3–12 μm). We suggest that SRH-DR5-B-iRGD specifically affects the growth of the smallest capillary network, which is supported by CD31 staining, where a significant reduction of small capillary network in SRH-DR5-B-iRGD-treated versus DR5-B-treated tumors was observed (Fig. 7A, C). Also, OA imaging may have certain advantages as it is also able to depict thrombosed vessels, whereas OCTA only detects the perfused vasculature. Overall, the combination of methods allows for a more comprehensive assessment and characterization of antiangiogenic effects.

Importantly, the anti-angiogenic properties of SRH-DR5-B-iRGD were consistent in two different xenograft models, namely, human glioblastoma U87MG and pancreatic cancer MIA PaCa-2. This indicates its potential versatility and suitability for treatment of various types of vascularized solid cancers. Considering that neo-angiogenesis plays a pivotal role in tumor growth, and that TRAIL-based drugs including SRH-DR5-B-iRGD are cytotoxic both towards tumor and endothelial cells, the anti-angiogenic role of SRH-DR5-B-iRGD is difficult to differentiate from its antitumor activity. These effects could potentially be better distinguished after a longer course of treatment, since the induction of apoptosis is a faster process compared to the suppression of angiogenesis.

## Conclusion

Using an integrated approach, the contribution of antiagiogenic SRH and iRGD moieties of fusion protein SRH-DR5-B-iRGD to the total antitumor effect was investigated in xenograft models of human glioblastoma and pancreatic cancer. The data obtained by multimodal microangiography were supported by molecular modeling and molecular biology techniques. Fusion of DR5-specific TRAIL variant DR5-B with anti-angiogenic peptides SRH and iRGD obviously resulted in significantly enhanced both anti-angiogenic and antitumor effects, suggesting the potential for further clinical application.

## Electronic supplementary material

Below is the link to the electronic supplementary material.

Supplementary Material 1

## Data Availability

The data generated and analyzed during the current study are available from the corresponding author on reasonable request.
