# Supplementary material for: Multivalent fusion protein targeting VEGFR2 and DR5 receptors: assessing the antiangiogenic and antitumor effects via multimodal microangiography
Source: J Transl Med. 2025 Aug 21;23:949. doi: 10.1186/s12967-025-06859-8 (PMC12372350; doi:10.1186/s12967-025-06859-8)
Supplement: Supplementary file 1 — Supplementary Material 1 [file 12967_2025_6859_MOESM1_ESM.pdf]

Supplementary Material

Multivalent fusion protein targeting VEGFR2 and DR5 receptors: assessing the antiangiogenic and antitumor effects via multimodal microangiography

Irina N. Druzhkova[1], Anna G. Orlova[2], Anastasiia S. Fedulova[3], Arina V. Avakiants[3,4], Alina A. Isakova[3,4], Ekaterina V. Kukovyakina[4], Yuan Zijian[3], Ekaterina A. Plotnikova[4,5], Galina V. Trunova[5], Andrey A. Pankratov[4,5], Anton A. Plekhanov[1], Alexey A. Kurnikov[2], Pavel V. Subochev[2], Alexey K. Shaytan[3], Marine E. Gasparian[4], Mikhail P. Kirpichnikov[3,4], Dmitry A. Dolgikh[3,4], Daniel Razansky[6,7*], Anne V. Yagolovich[3*]

[1] Privolzhsky Research Medical University, 603081 Nizhny Novgorod, Russia;

[2] A.V. Gaponov-Grekhov Institute of Applied Physics of the Russian Academy of Sciences, 603950 Nizhny Novgorod, Russia;

[3] Faculty of Biology, Lomonosov Moscow State University, 119234 Moscow, Russia;

[4] Shemyakin-Ovchinnikov Institute of Bioorganic Chemistry of the Russian Academy of Sciences, 117997 Moscow, Russia;

[5] P.A. Hertsen Moscow Oncology Research Institute - branch of the National Medical Research Radiological Centre of the Ministry of Health of the Russian Federation, 125284 Moscow, Russia;

[6] Institute of Pharmacology and Toxicology and Institute for Biomedical Engineering, Faculty of Medicine, University of Zurich, Switzerland;

[7] Institute for Biomedical Engineering, Department of Information Technology and Electrical Engineering, ETH Zurich, Switzerland

[*] Corresponding authors: Anne V. Yagolovich, Ph. D., yagolovichav@my.msu.ru, Daniel Razansky, Prof., daniel.razansky@uzh.ch

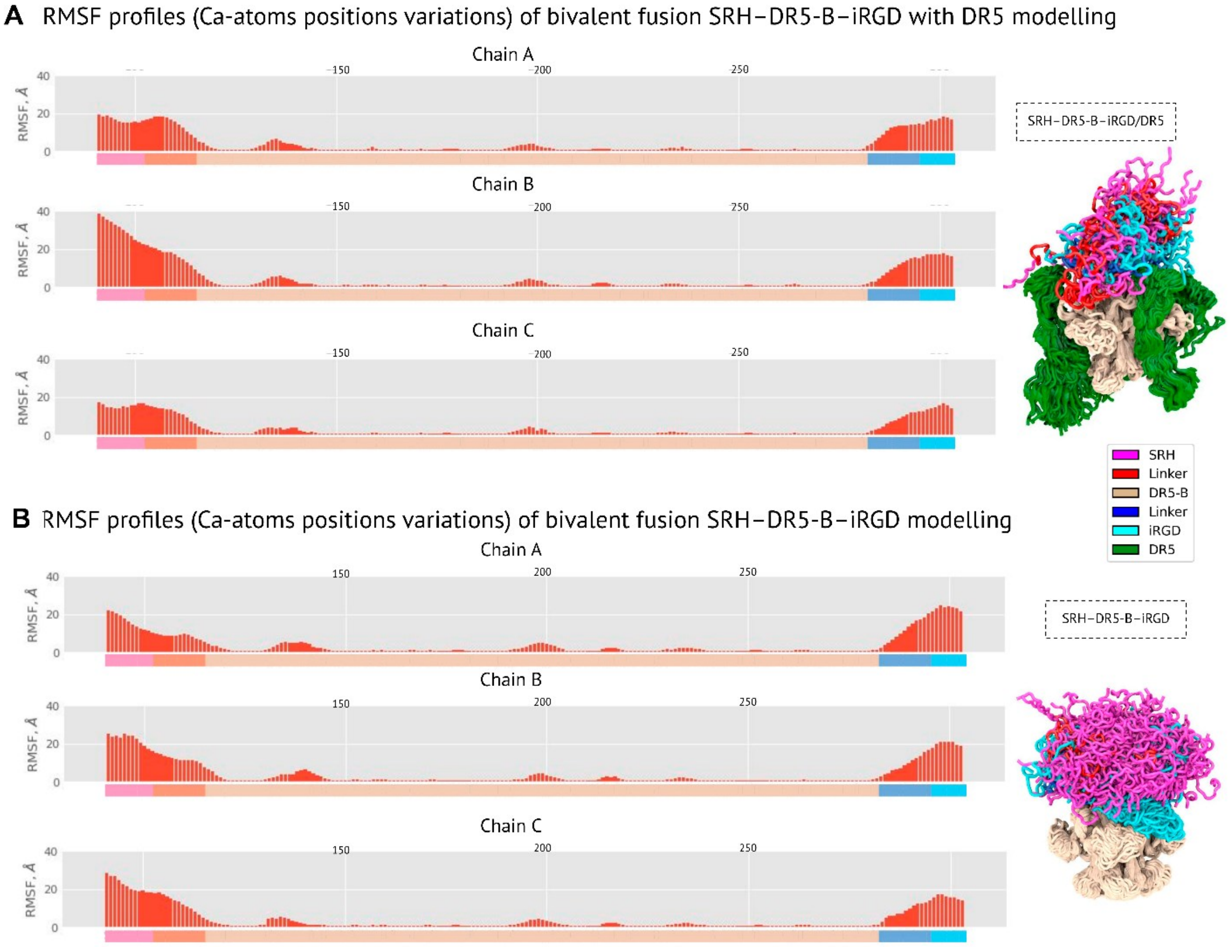

Fig. S1 Overview of the MD simulations. (**A-B**) RMSF profiles of Ca atom positions of SRH-DR5-B-iRGD molecules in MD simulation with DR5 (**A**) and without DR5 (**B**). An overview of the corresponding MD simulation trajectories is shown on the right.

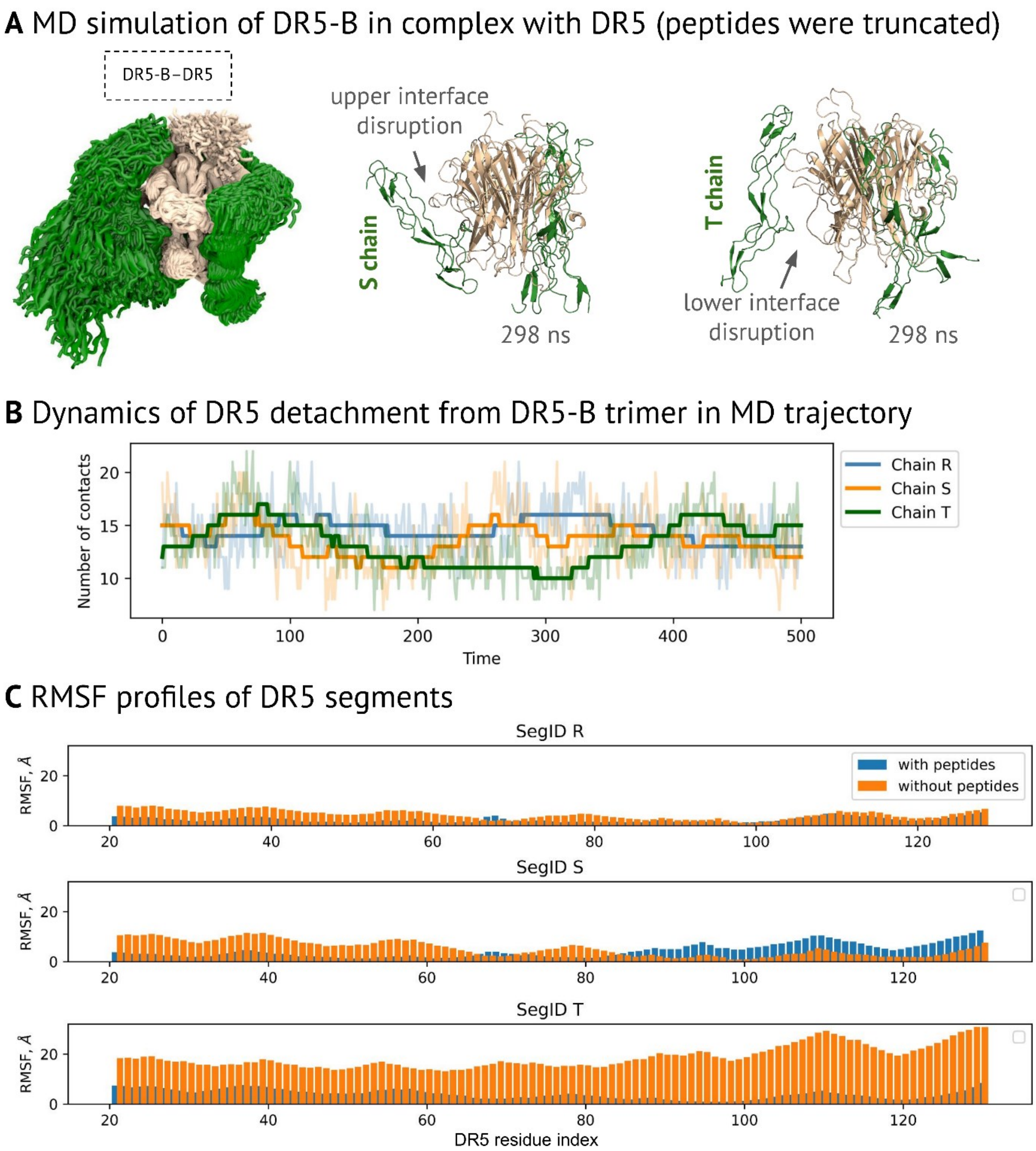

Fig. S2 Dissociation of DR5 from DR5-B upon truncation of SRH and iRGD peptides. (**A**) The MD simulation ensemble is shown as an overlay of MD trajectory frames and representative snapshots. (**B**) Dynamics of the number of contacts between DR5 and DR5-B in the MD simulation. (**C**) Comparison of RNSF values of DR5 segments between systems with and without peptides.

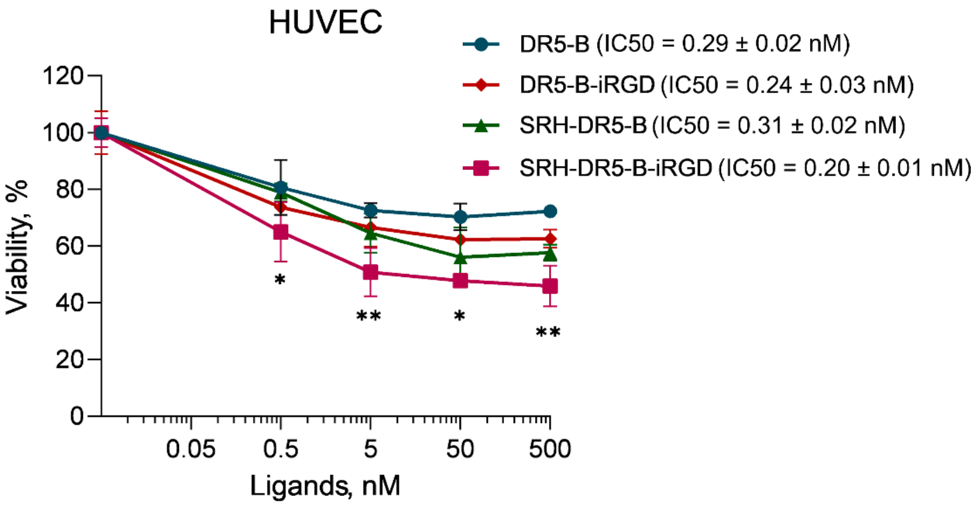

Fig. S3 Comparative assessment of the HUVEC viability after 48 h treatment with DR5-B, bispecific fusion proteins SRH-DR5-B and DR5-B-iRGD, or SRH-DR5-B-iRGD reveals the impact of each of SRH and iRGD effector peptides at the N- and C-termini of DR5-B, respectively. *$p < 0.05$, **$p < 0.01$ show the statistical significance of cell viability after SRH-DR5-B-iRGD compared with DR5-B, DR5-B-iRGD and SRH-DR5-B treatment. Two-way ANOVA with Tukey's multiple comparisons test.

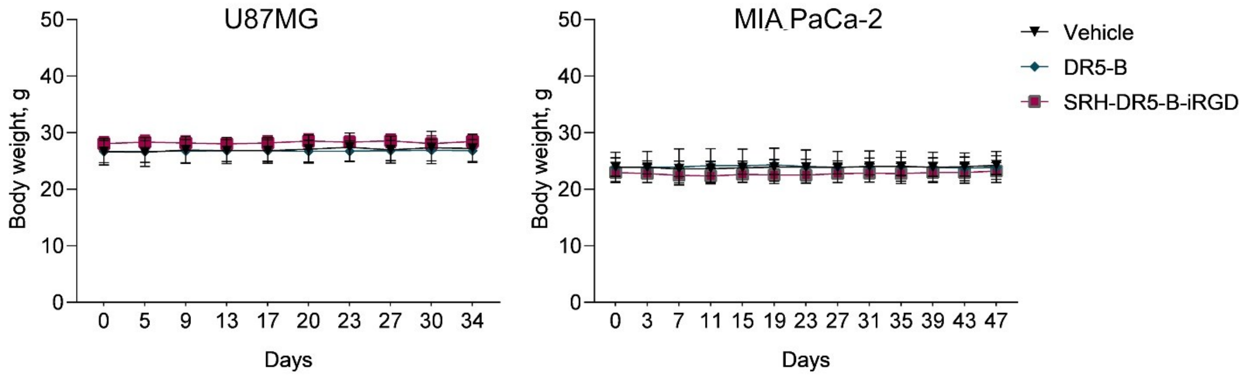

Fig. S4 Body weight of specific pathogene-free (SPF) NU–A/ATyrc/Tyrc Foxn1nu/Foxn1nu mice with xenografted tumors

Western blots

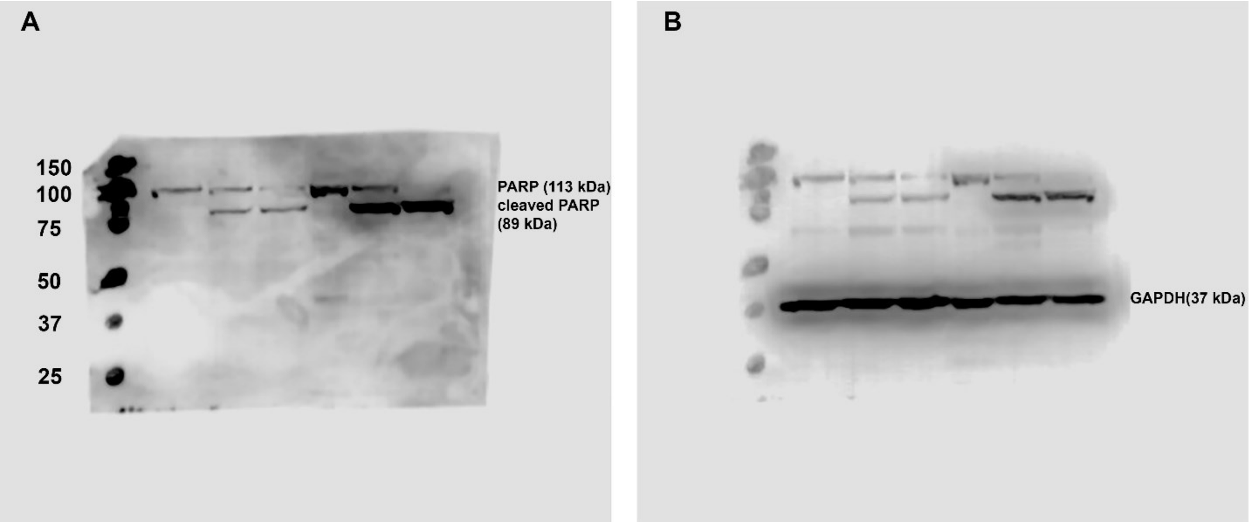

Fig. S5 (**A**) Staining with monoclonal antibodies to **PARP** (Invitrogen, clone 123) 1:500, Thermo Fisher Scientific, USA; (**B**) Staining with monoclonal antibodies to **GAPDH** (clone GA1R), 1:1000, Thermo Fisher Scientific, USA

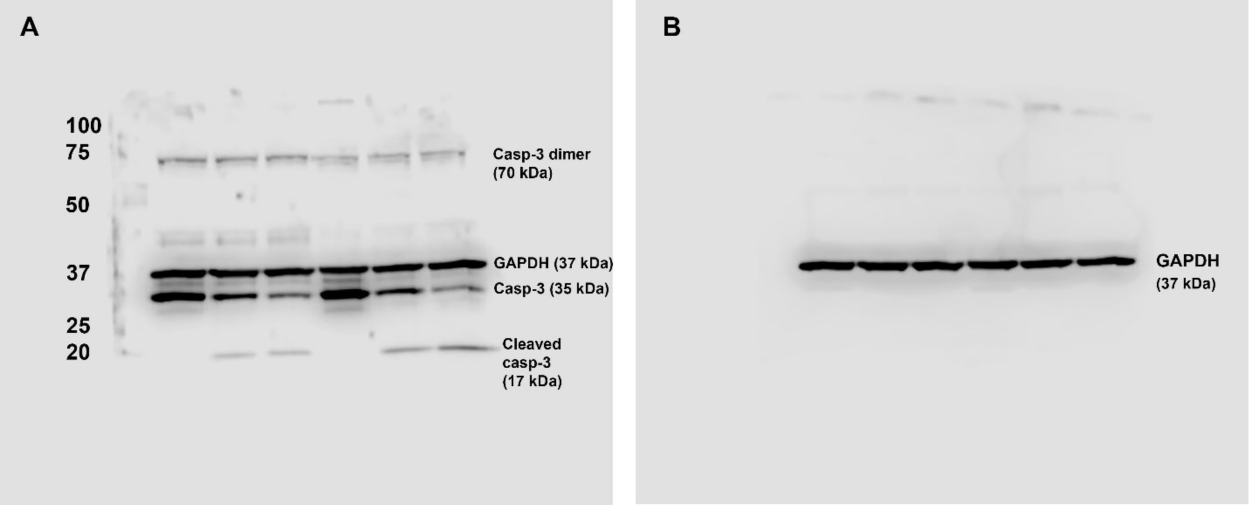

Fig. S6 (**A**) Staining with monoclonal antibodies to **caspase-3** (Cat. No. GTX110543), 1:1000, GeneTex, USA; (**B**) Staining with monoclonal antibodies to **GAPDH** (clone GA1R), 1:1000, Thermo Fisher Scientific, USA.

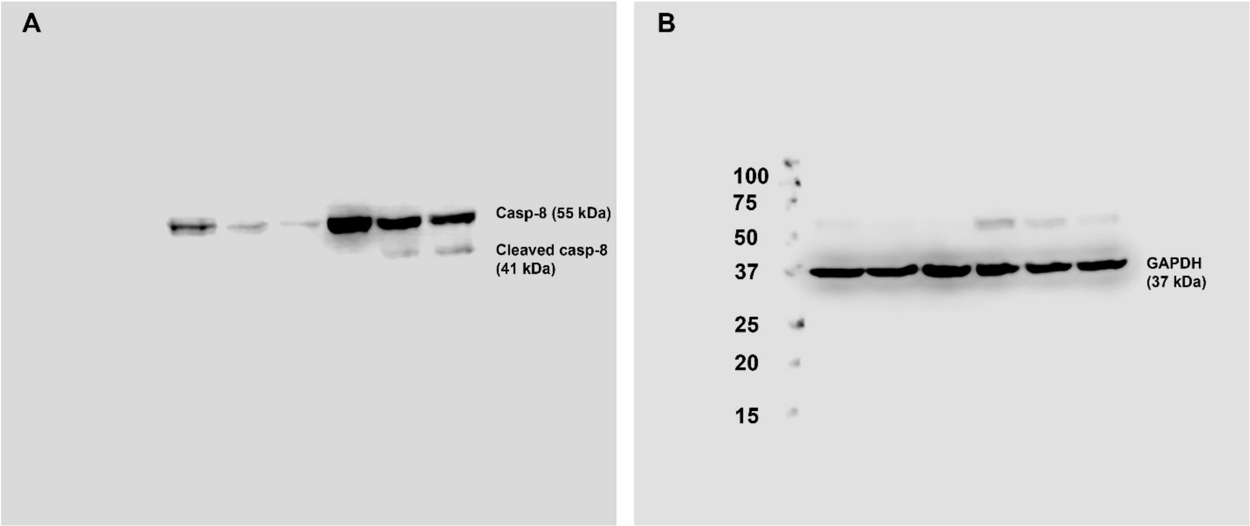

Fig. S7 (**A**) Staining with monoclonal antibodies to **caspase-8** (clone 5F7), 1:1000, Enzo Life Sciences, Farmingdale, NY, USA; (**B**) Staining with monoclonal antibodies to **GAPDH** (clone GA1R), 1:1000, Thermo Fisher Scientific, Waltham, MA, USA.

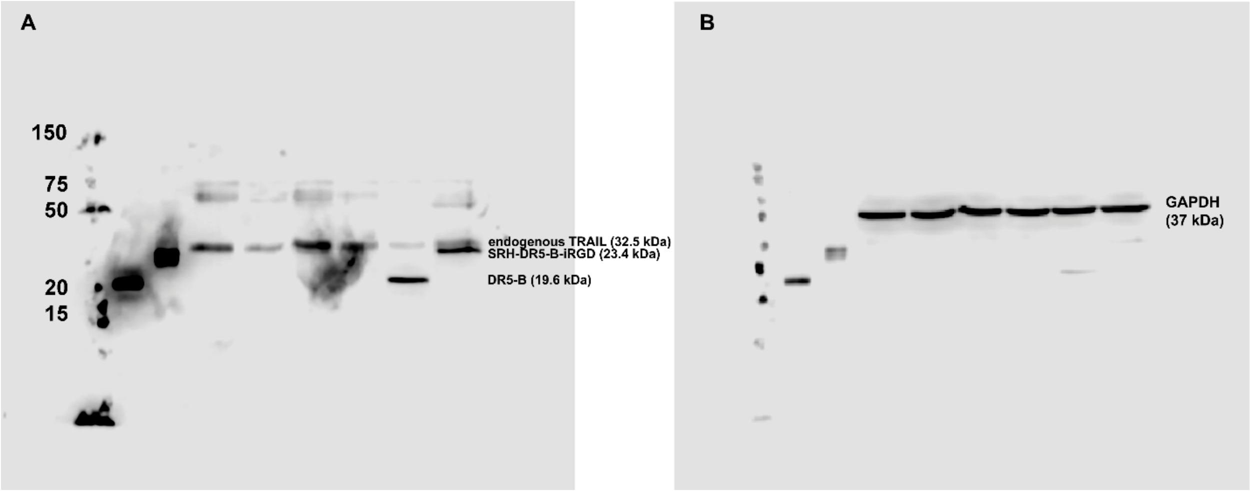

Fig. S8 (**A**) Staining with monoclonal antibodies to **TRAIL** (RRID AB_3074165), 1:1000, PeproTech, NJ, USA; (**B**) Staining with monoclonal antibodies to **GAPDH** (clone GA1R), 1:1000, Thermo Fisher Scientific, Waltham, MA, USA.
